# Harnessing Actinobacteria secondary metabolites for tuberculosis drug discovery: Historical trends, current status and future outlooks

**DOI:** 10.1007/s13659-025-00533-8

**Published:** 2025-08-11

**Authors:** Luana Layse Câmara de Almeida, Sayoane Pessoa Fernandes, Genil Dantas de Oliveira, Marcelly da Silveira Silva, Thalisson Amorim de Souza, Valnês S. Rodrigues-Junior, Samuel Paulo Cibulski

**Affiliations:** 1 Programa de Pós-Graduação em Ciências Farmacêuticas, Departamento de Farmácia, Universidade Estadual da Paraíba (UEPB), Campina Grande, Paraíba, Brazil; 2 Laboratório Multiusuário de Caracterização e Análise (LMCA), Instituto de Pesquisa em Fármacos e Medicamentos, Universidade Federal da Paraíba (UFPB), João Pessoa Paraíba, Brazil; 3 Programa de Pós-Graduação em Produtos Naturais e Sintéticos Bioativos, Universidade Federal da Paraíba (UFPB), João Pessoa, Paraíba, Brazil; 4 FACISA–Faculdade de Ciências da Saúde do Trairi, Universidade Federal do Rio Grande do Norte (UFRN), Santa Cruz, Rio Grande do Norte Brazil

**Keywords:** Antimycobacterial activity, Natural products, Actinomycete, Secondary metabolites, Antibiotics

## Abstract

Tuberculosis (TB) is a leading infectious disease killer and one of the major causes of deaths worldwide. Although TB is a curable and preventable disease, in 2023, approximately 10.8 million people fell ill with TB and there were an estimated 1.25 million of deaths worldwide. Despite some research progress for new drug candidates, drug repurposing, and new regimens, there is still an urgent need for the new medicins to treat TB, especially due to the growing cases of multidrug and extensively drug-resistant (MDR/XDR) strains. Drug resistance is a challenging obstacle to TB care and prevention globally, making TB harder and longer to treat, often with poorer outcomes for patients. The Actinomycetota encompass Gram-positive bacteria that produce a milieu of bioactive metabolites, including antibiotics, antiproliferative drugs, immunosuppressive agents, and other important medical molecules. Actinomycetota have a special place in the therapeutic arsenal to fight TB, as rifamycins, aminoglycosides, and cycloserine are derived from *Streptomyces* species, one of the most important genera in this phylum. Furthermore, hundreds of antimycobacterial metabolites have been isolated from Actinomycetota and can serve as effective drugs or useful agents for the discovery of new lead compounds to combat TB. The present review covers more than 171 isolated substances as potential antimycobacterial agents discovered between the years 1972 to 2024. Among the most potent compounds, with MIC in the submicromolar range, steffimycins, ilamycins/rufomycins, nosiheptide, actinomycins, lassomycin and boromycin are the most promising compounds. These compounds represent highly promising candidates for development of new antitubercular drugs. Additionally, some of these substances also demonstrated activity against resistant *Mycobacterium tuberculosis* (Mtb) strains, which is particularly relevant given the difficulty of treating MDR and XDR strains. Thus, actinobacteria have played and continue to play an important role in fight TB, remaining a promising source of antibiotic metabolites. Their unique metabolic diversity enables the production of metabolites with innovative mechanisms of action, making them a strategic reservoir for discovering therapies against untreatable forms of the disease.

**Graphical Abstract:**

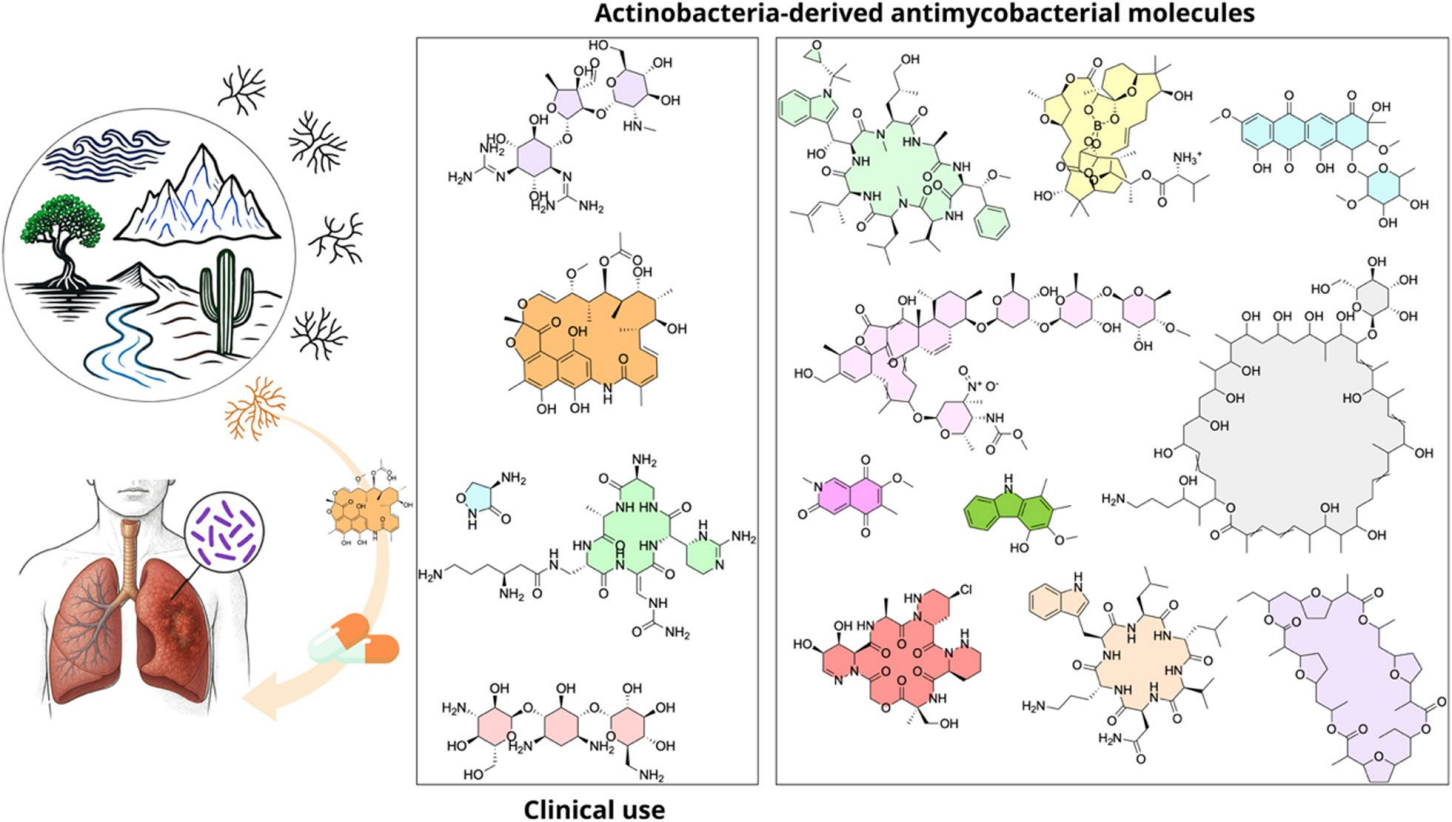

## Introduction

Since ancient times, products of natural origin have been fundamental for the discovery and development of medicines for the treatment of various diseases, mainly those caused by microorganisms, especially bacterial infections [1]. Notably, until the beginning of the twentieth century, approximately 80% of available medicines originated from plants [2]. It was only in 1928 that the discovery of penicillin by Alexander Fleming, from the fungus *Penicillium notatum*, marked a significant turning point in the history of natural products [3].

Natural products from microorganisms exhibit a wide range of biological properties, including antimicrobial, antitumoral, and immunosuppressive activities, as well as applications as veterinary drugs, pesticides, flavorings, and nutraceuticals [4]. Among these microorganisms, actinobacteria stand out, which are characterized by their high biosynthetic capacity to produce molecules of great clinical relevance [5].

Actinobacteria emerged approximately 2700 million years ago. They were initially obligate anaerobes, non-filamentous and non-spore-forming, in addition to presenting simple morphological characteristics [6]. Nowadays they are ubiquitous in Earth's major environmental compartments and their vast population sizes and rapid cellular production rates contribute to an extraordinary genetic diversity [7]. Most members of this phylum have a complex lifestyle, forming aerial and substrate mycelium, [8, 9].

Currently, they are one of the most diverse groups of microorganisms found in nature, characterized by a peptidoglycan-rich cell wall, Gram-positive, with high content of guanine and cytosine in their genome and are widely distributed in different habitats, including the microbiomes of higher eukaryotic organisms [10–12]. When resources are scarce, these microorganisms produce aerial hyphae that segment themselves, giving rise to spores that are capable of withstanding adverse conditions and easily dispersing to new environments or nutrient sources [13].

The phylum represents one of the largest phyla in the Bacteria domain, with the *Streptomyces* genus being the largest [14]. The genus *Streptomyces* has more than 800 species with validated nomenclature, being responsible for approximately 39% of all described microbial metabolites, including about 80% of known bioactive molecules [15, 16]. The production of these bioactive compounds is directly related to the nutritional and environmental conditions of the habitat. These compounds are often classified as stress metabolites, since they are produced as an adaptive response to adverse environmental conditions, aiding in survival and competition with other microorganisms [17].

The remarkable genetic potential of these microorganisms for the production of a wide variety of secondary metabolites is evidenced by the presence of numerous biosynthetic gene clusters (BGC) in their genomes. Investigation of the metabolic potential of the genus *Streptomyces* revealed a vast set of modular biosynthetic genes, responsible for the production of several complex secondary metabolites [18–20]. They usually produce antibiotics using large enzyme complexes, such as polyketide synthases, non-ribosomal peptide synthases or a combination of both, in which these multienzyme complexes perform different chemical alterations, and thus there is a wide production and variety of antibiotics [13]. However, isolates belonging to the same species but originating from different environments may present distinct repertoires of these BGC related to the biosynthesis of bioactive compounds, reflecting great genomic flexibility and adaptation to specific ecological conditions [21].

Currently, in the genomic era, the integration of genomics, chemical dereplication, and the exploration of novel environments/habitats has become a pivotal strategy in the search for antimicrobial compounds from microorganisms, including Actinobacteria. Advances in genome mining and metagenomics enable the rapid identification of BGC encoding potential antimicrobial metabolites, while chemical dereplication techniques streamline the identification and elimination of known compounds, focusing efforts on discovering novel scaffolds [22]. By targeting underexplored ecological niches, such as extreme environments, marine sediments, and plant microbiomes, researchers can uncover unique microorganisms with untapped biosynthetic potential. This triad, that comprehends genome mining, chemical dereplication and underexplored ambient, not only accelerates antibiotic discovery but also enhances the efficiency of isolating structurally diverse and biologically active molecules to combat rising antimicrobial resistance.

The identification of streptomycin as the first successful antibiotic to treat TB from Actinobacteria stimulated intensive exploration of these filamentous microrganisms, leading to the discovery of a wide variety of bioactive compounds [23]. Despite the scientific advances achieved over the years, there has been a significant decrease in the discovery of new natural products, evidenced by the fact that approximately 50% of currently available antimicrobials were identified during the so-called “Golden Age” of antibiotics, between the 1950s and 1960s [4]. At the same time, even with continuous efforts to control and treat TB, the emergence of drug-resistant strains remains a relevant challenge for global health [24].

## *Mycobacterium tuberculosis* and tuberculosis (TB)

Tuberculosis (TB) is a communicable disease that affects millions of people worldwide and returned to be the leading global cause of death caused by a single infectious agent, replacing COVID-19 [25]. This pathology stands out for mainly affecting the lungs, causing pulmonary TB. However, TB can affect other organs, resulting in extrapulmonary TB [26, 27]. The pulmonary form of the disease accounts for approximately 85% of cases reported globally and is considered a major contributor to antimicrobial resistance [28].

TB is transmitted through the dissemination of aerosols in the environment when an infected individual cough, speaks, or sneezes [27]. Once inhaled, Mtb travel through the respiratory system until they reach the lungs, where innate immune mechanisms are activated, allowing alveolar macrophages to engulf the infecting bacilli and attempt to destroy them through proteolytic enzymes and cytokines, which can lead to the elimination of the microorganism or the formation of granulomas [29]. Mtb can remain in granulomas in its latent and asymptomatic form for many years. However, this infection can be reactivated, resulting in the active form of the disease, characterized by being transmissible and contagious [30].

TB is believed to have existed for thousands of years, with the first written records of the disease dating back to 3300 and 2300 years ago [31]. The Hippocratic school considered TB, known at the time as phthisis, a disease of hereditary origin, not recognizing its contagious nature [32]. The possible contagious etiology of the disease was initially suggested by Isocrates and, later, by Aristotle, who, when describing cases of cervical lymphadenopathy compatible with scrofula in domesticated pigs and cattle, indicated transmission through inhalation of contaminated aerosols, referring to the process as a result of exposure to “polluted air”. Galen, later, also demonstrated adherence to the contagious theory, recommending avoiding close contact with individuals affected by the disease [33]. Over the years, despite advances in the understanding of various pathological and clinical aspects of TB, its etiopathogenesis remained unknown [34].

It was only in 1882 that the causative agent of TB was identified, thanks to Robert Koch who in a short time managed to isolate the microorganism and reproduce the disease in experimental animals [35, 36]. In the early twentieth century, Koch's collaborator Carl Flügge suggested that the tubercle bacillus was spread to new individuals through microscopic respiratory particles, showing that exposure to these droplets expelled by tuberculous patients could result in infection [33].

Mtb is a rod-shaped bacterium, immobile, grows slowly, and is generally aerobic, but is capable of surviving in hypoxic environments during latent infection. In addition, it has an important component in its cell wall, mycolic acid, which gives it the acid-fast characteristic [37]. It is characterized by being a bacterium with a versatile metabolism, being able to switch to alternative metabolic pathways when exposed to drugs or stress, which allows it to withstand long periods of dormancy [38], being able to persist in a latent state in most infected individuals without causing evident clinical manifestations [39]. In addition, it uses proteins similar to eukaryotic proteins to manipulate host signaling pathways, favoring its survival and intracellular replication [40].

Regarding TB prevention, BCG (Bacillus Calmette-Guérin), first administered to humans in 1921, is one of the most widely used vaccines globally and remains an integral part of national immunization programs for TB prevention in many countries [41, 42]. This vaccine is composed of an attenuated strain of *M. bovis* and offers protection against Mtb. BCG is known to induce trained immunity, a mechanism triggered by exogenous or endogenous stimuli that results in an enhanced effector function of immune cells after a second exposure, ensuring a more efficient response [43].

BCG has a well-established safety profile, presenting immunomodulatory effects that make it particularly effective against central nervous system TB and disseminated TB when administered at birth or in school-aged children. It remains a standard vaccine that demonstrates efficacy comparable to the most recent subunit TB vaccines tested to date [44]. The vaccine is routinely administered shortly after birth in countries with a high TB burden, with the aim of preventing severe forms of the disease [45]. Since its introduction in Europe in the 1920s, the BCG vaccine has been associated with a significant reduction in infant mortality to a magnitude that exceeds the expected effect of specific prevention against TB. Of note, much of this reduction is observed during the first year of life, particularly among infants who received the immunization [44, 46]. New vaccines for TB are being developed aiming to be safer and more effective than the BCG vaccine. In 2024, there were fifteen new vaccine candidates under clinical investigation, six of which were in Phase III trials [25].

## Tuberculosis treatment: past, present and, future

Before the emergence of antibiotics for the treatment of TB and, even with the advances in the diagnosis of this pathology in the nineteenth century, therapies were still immature. The therapeutic regimen indicated for that time was based on Galenic principles, including staying in places with a temperate climate, isolation, good hygiene and nutrition [34]. Even before the twentieth century, numerous surgical techniques were developed for the control and treatment of TB, such as pneumothorax, lung collapse, cavity drainage, thoracoplasty and lung’s resection [47].

In 1943, Selman Waksman isolated the *Streptomyces griseus*, the microorganism responsible for the production of streptomycin. Thus, the first effective antibiotic in the treatment of TB emerged, resulting in a significant reduction in lesions caused by the disease, and a clear drop in mortality [48, 49]. Its use expanded rapidly, leading to the early emergence of resistance mechanisms [50].

Shortly after the introduction of streptomycin, new medications were added for TB treatment, notably ρ-aminosalicylic acid, thiosemicarbazone and isoniazid [51]. It was observed that the administration of these agents together with streptomycin resulted in an increased cure rates [52]. However, despite these advances, side effects, drug resistance and the high number of new cases stimulated the search for new pharmacological treatments [53].

**Fig. 1:**
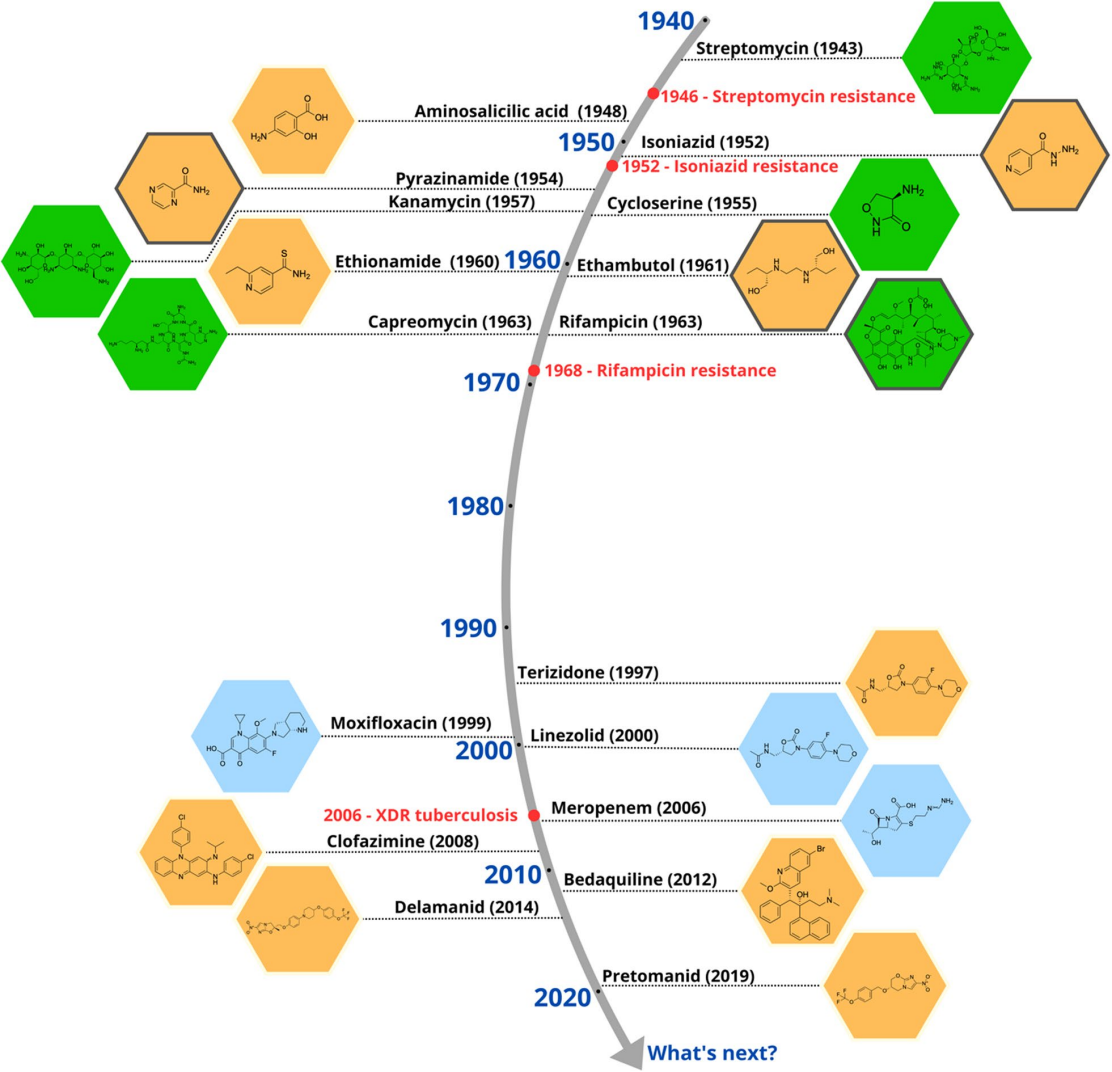
Milestones in the discovery and use of drugs against Mtb. The golden age of anti-tubercular drug discovery (1940–1960) saw transformative breakthroughs, revolutionizing TB treatment and reducing mortality. The subsequent 40-year drought (1970–2000) stalled progress due to waning industry investment, reliance on existing drugs, and the rise MDR-TB lineages

In 1957, rifamycin, a potent antibiotic against Mtb was isolated from *Streptomyces mediterranei* [54]. By the late 1960s, its derivative rifampicin was introduced into clinical practice, becoming a cornerstone in the treatment of patients with TB, particularly those who had developed resistance to previously available therapies [55, 56]. In subsequent years, additional antimycobacterial agents with complementary mechanisms of action were identified, including kanamycin [57], capreomycin [58], and cycloserine [59]. These drugs have played, and continue to play, a critical role in in the treatment of TB, especially in multidrug-resistant TB (MDR-TB) [60]. Figure 1 outlines the introduction and evolution of antitubercular drugs used in clinical practice.

Currently, rifampicin is one of the main drugs in the first-line treatment regimen for drug-sensitive TB. This treatment consists primarily of the combination of 4 drugs, rifampicin, isoniazid, ethambutol and pyrazinamide for a period of 2 months, followed by isoniazid and rifampicin for an additional period of 4 months [27, 61]. In 2023, treatment success for drug-susceptible tuberculosis remained high, with an effectiveness rate of 88% [25]. On the other hand, with 10.8 million new cases worldwide, TB incidence has only decreased by 8.3% from 2015 to 2023, falling far short of the End TB Strategy's 50% reduction target for 2025 [25].

## Drug-resistant TB: definitions, global burden, and therapeutic advances

According to the WHO, drug-resistant TB is classified into five categories: isoniazid-resistant TB, rifampicin-resistant TB (RR-TB), MDR-TB (resistant to rifampicin and isoniazid), pre-XDR-TB (resistant to rifampicin and any fluoroquinolone), and XDR-TB (resistant to rifampicin, plus any fluoroquinolone, plus at least one of either bedaquiline or linezolid) [25, 62]. These distinct resistance profiles underscore the urgent need for tailored treatments and intensified efforts in surveillance, diagnosis, and drug development.

Drug-resistant TB continues to be a public health threat and is among the 24 pathogens that have been incorporated into the WHO Bacterial Priority Pathogens List [25]. Drug resistance is a major challenge in several regions of the world, with estimates indicating that drug-resistant TB could generate a global economic burden of approximately US$ 16.7 trillion between 2015 and 2050, accounting for 20% and 25% of the projected total cost of antimicrobial resistance during that period [62]. In 2023, worldwide healthcare systems identified and provided treatment for 175,923 individuals with MDR-TB. However, this represents just 44% of the estimated 400,000 people who developed these drug-resistant forms of TB that year [25]. This substantial diagnostic and treatment gap reflect ongoing challenges in access to healthcare services, particularly in low- and middle-income countries.

In addition to genetic mutations and intrinsic mechanisms (not discussed in this review), several other factors have been implicated in the development of resistance to TB treatment. These include interindividual differences in drug metabolism, activation of efflux pumps, poor drug penetration into TB-infected tissues, low-quality pharmaceutical formulations, suboptimal dosing, limited access to healthcare services, insufficiently trained healthcare personnel, as well as socioeconomic determinants such as poverty and overcrowding. Collectively, these elements contribute to treatment failure and the selection of resistant strains. These insights underscore the need for comprehensive strategies encompassing the development of novel therapeutic agents, optimized drug delivery systems, and broader public health interventions [62, 63]. Notably, TB treatment becomes more challenging with the emergence of resistant variants, as patients with resistance to first-line treatments often require prolonged regimens involving second-line drugs, which are generally less effective, more toxic, and more expensive [64, 65].

WHO has established all-oral treatment protocols for MDR/RR-TB, representing a significant improvement over previous injectable-based regimen. Currently, WHO recommends three distinct categories of treatment for drug-resistant TB. The first category features two all-oral 6-month treatment protocols for individuals with MDR/RR-TB, applicable regardless of fluoroquinolone resistance status. The second category encompasses various 9-month all-oral protocols specifically designed for MDR/RR-TB patients without fluoroquinolone resistance. The third category consists of extended 18–20-month regimens that may incorporate an injectable medication (amikacin). The 6-month protocols are considered preferable, while the longest regimens are reserved as a final option. Importantly, recent years have shown substantial improvement in treatment outcomes for MDR/RR-TB patients. For those beginning treatment in 2021 (the most recent year with complete outcome data), the success rate reached 68%, though this remains considerably lower than rates achieved with drug-sensitive TB [25]. The spread of these resistant strains has become a major concern for public health, making it urgent to discover new drugs with different mechanisms of action that reduce the duration of TB treatment and are not impacted by pre-existing resistance [66, 67].

Advancing TB research and innovation is critical, with a focus on developing less complex and quicker treatment regimens. The WHO has established a comprehensive global strategy to enhance and expedite TB research efforts while ensuring fair access to research benefits. Promising developments are emerging in new diagnostic tools, medications, and vaccines for tuberculosis [25]. As of August 2024, the pipeline for TB treatment medications had expanded to 29 drugs undergoing clinical trials (Phase I, II, or III), representing significant growth from just eight candidates in 2015. The portfolio includes 18 novel chemical entities: alphibectir (BVL-GSK098), BTZ-043, delpazolid, GSK-286, ganfeborole (GSK-3036656), macozinone, MK-7762 (TBD09), quabodepistat (OPC-167832), TBAJ-587, TBAJ-876, TBI-223, pyrifazimine (TBI-166), TBA-7371, telacebec (Q203), sanfetrinem, SQ109, sutezolid, and sudapyridine (WX-081). Additionally, the pipeline features three WHO-approved treatments (bedaquiline, delamanid, and pretomanid) alongside eight repurposed medications: clofazimine, levofloxacin, linezolid, moxifloxacin, high-dose rifampicin, rifapentine, sitafloxacin, and tedizolid. Beyond individual drugs, researchers are also evaluating various combination regimens incorporating both new and repurposed compounds, as well as host-directed therapies, through Phase II, Phase III/IV trials, and operational research initiatives [25].

## Actinomycetota: nature's molecular architects–an unparalleled reservoir of antimycobacterial metabolites

### Mapping the scientific output on actinobacteria and TB drug discovery

**Fig. 2:**
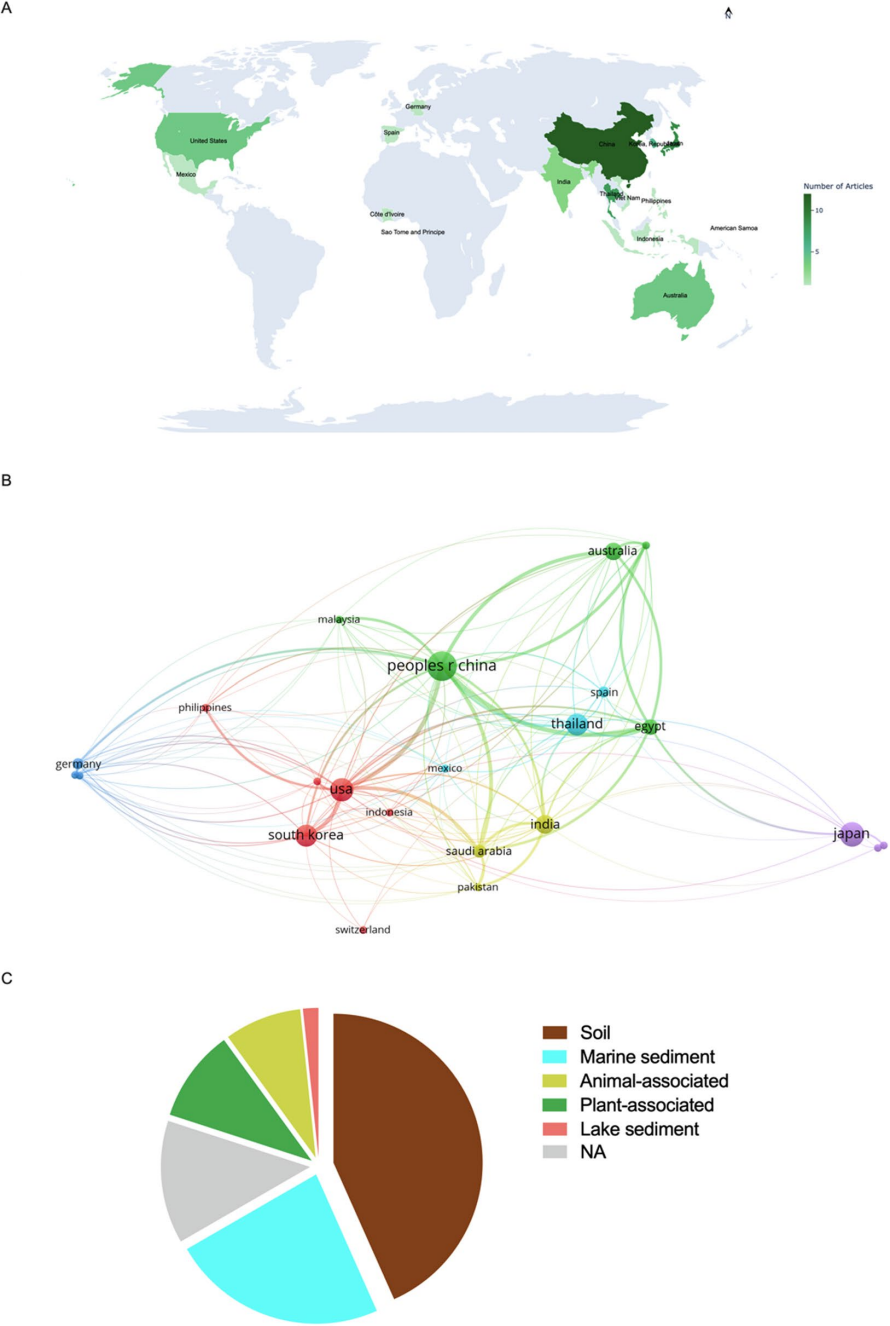
Bibliometic data and Actinobacteria-producing antimycobacterial metabolites source. **A** Number of studies conducted on the isolation of antimycobacterial metabolites by country. **B** VOSviewer network visualization of the selected studies. **C** Distribution of actinobacteria-derived metabolites with antimycobacterial properties based on bacterial habitat (isolation source)

As already mentioned, the compounds produced by Actinobacteria have a long history of use in the treatment of TB. From this great asset, many molecules with antimycobacterial activity were discovered. However, the activity of the vast majority of these molecules was not explored clinically, or even in animal tests. In this context, the present work provides an overview of natural substances with antimycobacterial activity derived from Actinobacteria. This review, that compiles 171 molecules with antimycobacterial activity, was performed thorough in the PubMed database using the terms: “Actinobacteria", "Actinomycete", "*Streptomyces*" and "*Mycobacterium tuberculosis*”. Three hundred eighty-four articles were retrieved, after removing duplicates and articles that not present elucidated chemical structures, 60 articles published between 1972 to 2024 were elected to deep analyses. Figure 2 depicts a bibliometric data extracted from the selected articles.

Of the sixty articles retrieved, the most were from Asia (68.3%), with China, Japan and Thailand having the largest contributions (29 publications, 48.3%) (Fig. 2A). Figure 2B displays the geographical distribution of the selected publications and the connections between different nations. The analysis was performed in the VOSviewer software. VOSviewer is a widely used tool for bibliometric and network analysis, enabling clear and interactive visualization of scientific data [68]. Thus, the figure helps identify the main collaborating countries and their impact on the scientific production related to the topic reviewed in this work.

The profile displayed in Fig. 2A, B demonstrates a significant concentration of scientific output in Asia, underscoring the region's leading role in research and development of novel antimicrobial agents derived from *Streptomyces*. These findings are consistent with those of Leite et al. [69], who identified Asia as the region with the highest number of patents related to bioactive compounds produced by *Streptomyces* species with antimicrobial activity. This could be related to public policies that foster technological innovation, alongside a well-established tradition in industrial microbiology and microbial bioprospecting [69].

Although microbial diversity is globally distributed, scientific advances in Actinobacteria research are largely concentrated in countries with robust infrastructure and funding. China has become a global leader in scientific output due to strategic investments in science and technology, particularly in biotechnology and public health [70]. Similarly, Japan’s prominence stems from a strong post-war tradition in antibiotic discovery and the work of key research institutions dedicated to novel antimicrobials [69].

### Ecological origins of actinobacteria-derived metabolites

Regarding the sources of isolation of antimicrobial-producing actinobacteria, the presence of these microorganisms was observed in diverse environments, with emphasis on soil (40%), marine sediments (23%), plants (10%) and, animals (8%) (Fig. 2B). This can be attributed to the high complexity of terrestrial environments [71]. However, in recent decades, a growing number of studies have shown that obtaining new species of *Streptomyces* from terrestrial environments has become progressively more limited, with the rediscovery/reisolation of secondary metabolites already known to be produced by these bacteria being frequent [72]. In this context, actinobacteria from marine ecosystems have stood out for their remarkable biosynthetic potential. This chemical diversity is strongly influenced by the adverse physical–chemical conditions of these habitats, including extreme variations in pressure, salinity, light, and temperature [73, 74]. Furthermore, bioactive compounds obtained from marine microorganisms have proven to be particularly promising, due to the greater probability of presenting novel chemical structures and relevant pharmacological activities [75].

Several microbial communities have been identified within the internal tissues of plants as endophytes, which perform ecologically significant functions in the plant environment, being recognized for their ability to produce compounds that promote plant growth, act as repellents against insects and phytopathogens, and contribute to the tolerance of adverse abiotic stress conditions [76, 77]. In addition, actinobacteria have increasingly been recognized for their symbiotic associations with eukaryotic hosts, being widely distributed both on the external surfaces and in the digestive tract of various animals. Interestingly, certain insects show a functional dependence on these bacteria for nutritional supplementation, highlighting their ecological and functional importance in mutualistic relationships [6, 7]. Thus, actinobacteria continues to represent a promising yet underexplored reservoirs of bioactive compounds.

### Classification and overview of antimycobacterial metabolites from actinobacteria

**Fig. 3:**
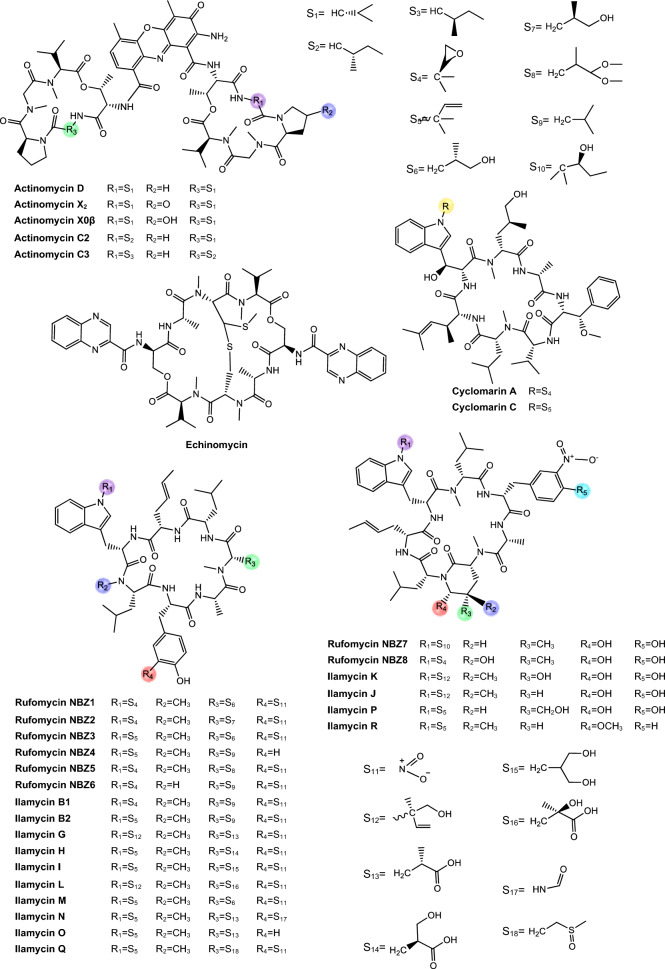
Chemical structures of antimycobacterial NRP metabolites

**Fig. 4:**
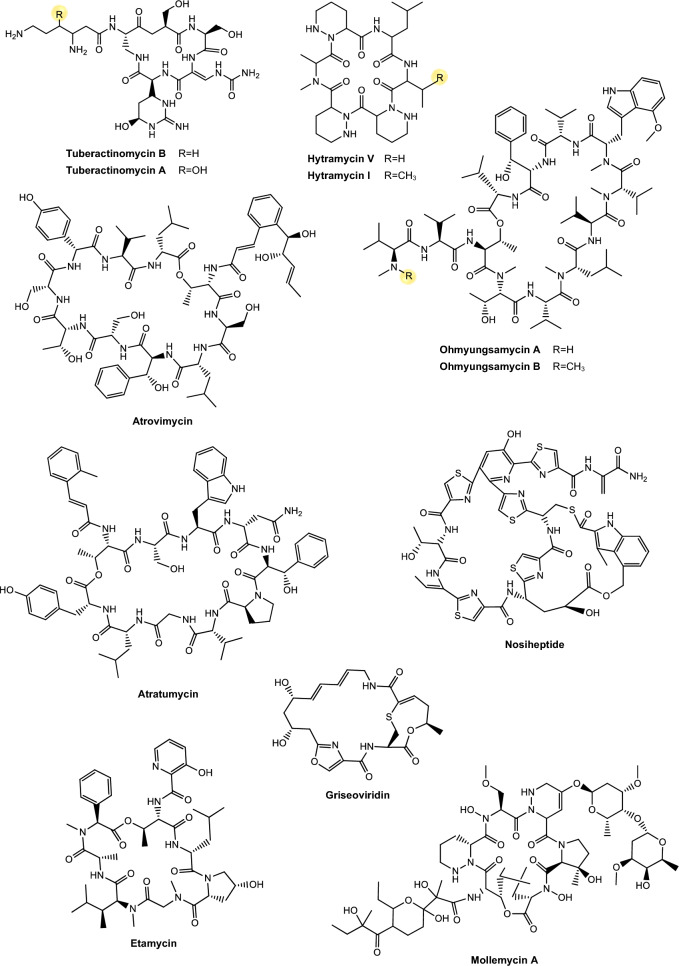
Chemical structures of antimycobacterial NRP metabolites

**Fig. 5:**
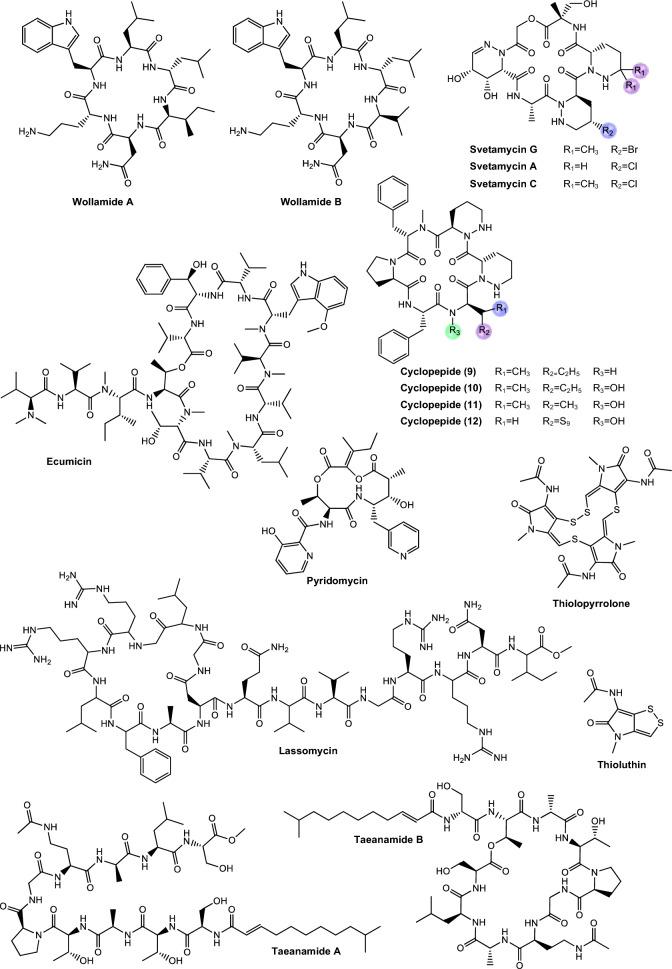
Chemical structures of antimycobacterial NRP metabolites

**Fig. 6:**
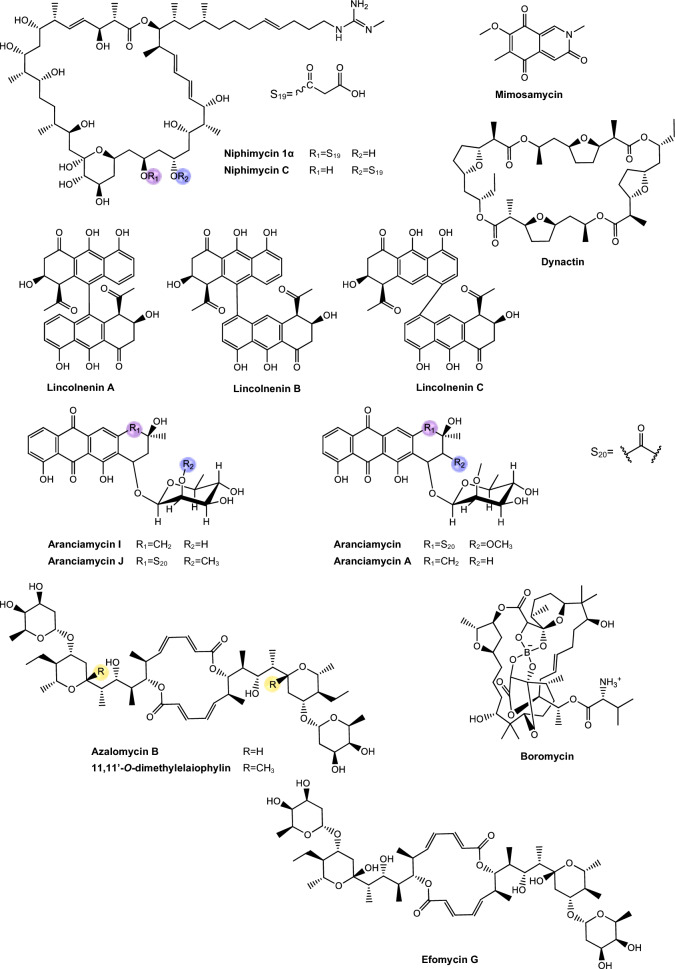
Chemical structures of PKS-derived antimycobacterial metabolites

**Fig. 7:**
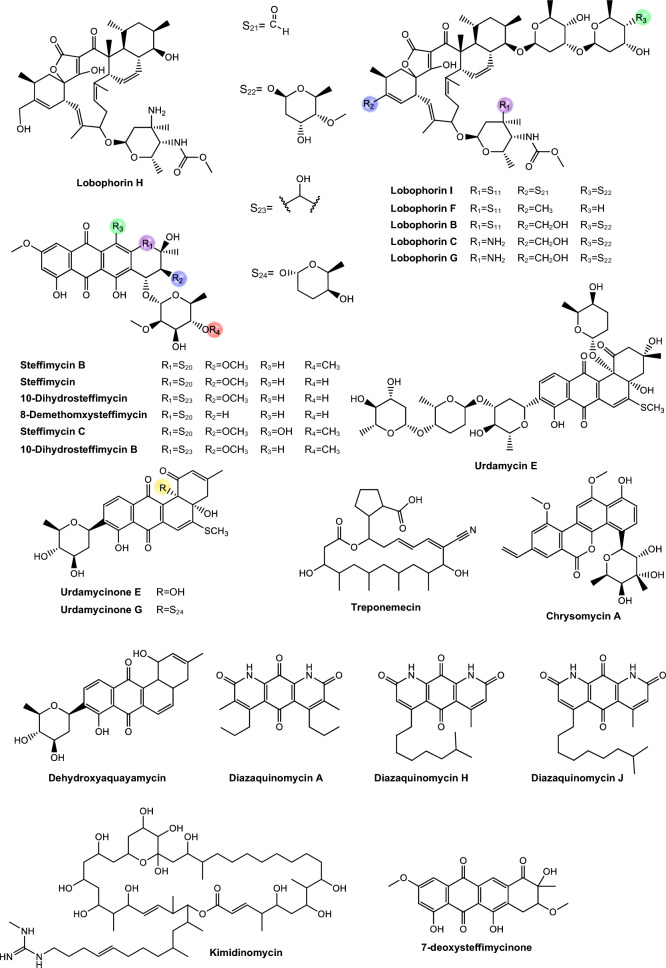
Chemical structures of PKS-derived antimycobacterial metabolites

Regarding antimycobacterial potential of the retrieved metabolites, the compounds were classified based on the minimum inhibitory concentration (MIC) values, being categorized as follows: compounds with MIC > 10.0 μM were considered to have weak activity, those with MIC between 1.0 μM and 10.0 μM were classified as having moderate activity, while compounds with MIC < 1.0 μM were classified as potent. All the substances analyzed are listed in Tables 1, 2, 3. The compiled data presents the Actinobacteria specie, isolation source, local/country of isolation, and MIC, in μg/mL and μM. The chemical structures were drawn in the ChemDraw software 23.1.2 and are presented in Figs. 3, 4, 5, 6, 7, 10, 11. For the purposes of in-depth analysis and discussions, we selected only the compounds that demonstrated moderate and potent activity, since these may have greater pharmacological relevance in the context of the development of new antitubercular agents.

**Table 1:** NRP from Actinobacteria with antimycobacterial activity

Metabolite	Actinobacteria specie	Country^a^	Source	Microorganism-test	MIC (μM)	MIC (μg/mL)	Reference
Actinomycin D	*Streptomyces griseoruber* NBRC 12873	IN	Soil	Mtb H37Rv	0.62	0.78	[82]
Actinomycin X_2_	*Streptomyces smyrnaeus *UKAQ_2	SA	Mangroove	Mtb H37Ra	1.23	1.56	[83]
				*M. bovis* BCG	1.23	1.56	
				Mtb H37Rv	2.08	2.64	
Actinomycin D				Mtb H37Ra	1.24	1.56	
				*M. bovis* BCG	1.24	1.56	
				Mtb H37Rv	1.43	1.8	
Actinomycin X0β	*Streptomyces avermitilis *MS449	CN	Marine sediment	*M. bovis *BCG	0.20	0.25–0.5	[84]
				Mtb H37Rv	6.29	8.0	
Actinomycin X_2_				*M. bovis* BCG	0.20	0.25–0.5	
				Mtb H37Rv	0.79	1.0	
Actinomycin D				*M. bovis *BCG	0.20	0.25–0.5	
				Mtb H37Rv	6.37	8.0	
Actinomycin C1	*Streptomyces* sp. IIIM06	IN	Soil	Mtb H37Rv	0.05	0.0625	[85]
Actinomycin C2					0.03	0.039	
Actinomycin C3					0.03	0.039	
Actinomycin D	*Streptomyces* sp. InaCC A758	ID	Soil	Mtb H37Rv	0.62	0.78	[86]
Cyclomarin A	*Streptomyces* sp. MM334-153F1	JP	Soil	Mtb H37Rv	0.48	0.5	[87]
				Mtb XDR*-Mtb*5	1.92	2.0	
				Mtb XDR*-Mtb*44	7.19	7.5	
				*M. smegmatis*	0.45	0.47	
Cyclomarin C	*Streptomyces* sp. BCC26924	TH	Soil	Mtb H37Ra	0.10	0.10	[88]
Echinomycin	*Streptomyces *sp. LS462	CN	Soil	*M. bovis *BCG	0.09	0.1	[89]
				Mtb H37Rv	0.45	0.5	
Rufomycin NBZ1	*Streptomyces atratus* MJM3502	KR	Soil	Mtb H37Rv	0.25		[90]
Rufomycin NBZ2					0.44		
Rufomycin NBZ3					0.84		
Rufomycin NBZ4					> 10		
Rufomycin NBZ5					0.11		
Rufomycin NBZ6					0.57		
Rufomycin NBZ7					0.10		
Rufomycin NBZ8					0.03		
Rufomiazine					> 10		
Ilamycin B2					0.2		
Ilammycin B1					1.7		
Epimeric mixture C-32 RUFs I and II					0.02		
Ilamycin G	*Streptomyces atratus* SCSIO	CN	Marine sediment	Mtb H37Rv	9.5		[91]
Ilamycin H					9.5		
Ilamycin I					1.2		
Ilamycin J					0.0096		
Ilamycin K					1.4		
Ilamycin L					0.24		
Ilamycin M					2.3		
Ilamycin N					9.6		
Ilamycin O					10.0		
Ilamycin P					1.4		
Ilamycin Q					1.4		
Ilamycin R					1.2		
Tuberactinomycin A	*Streptomyces griseoverticillatus*	JP	Soil	Mtb ATCC 607	17.81	12.5	[92]
Tuberactinomycin B					4.67	3.2	
Hytramycin V	*Streptomyces hygroscopicus* ECUM 14046	NA	NA	Mtb H37Rv	17.84	11.3	[93]
				Main Mtb global clades	14.68–36.15	9.3–22.9	
				Mtb resistant strains: streptomycin (rSM, ATCC 35820), rifampicin (rRMP, ATCC 35838)	15.16–18.32	9.6–11.6	
Hytramycin I				Mtb H37Rv	9.27	6.0	
				Main Mtb global clades	6.80–7.42	4.4–4.8	
				Mtb resistant strains: streptomycin (rSM, ATCC 35820), rifampicin (rRMP, ATCC 35838)	7.26	4.7–4.7	
Atrovimycin	*Streptomyces atrovirens* LQ13	CN	Soil	Mtb H37Rv	1.88	2.5	[94]
Ohmyungsamycin A	Actinobacteria strain SNJ042	KR	Soil	Mtb H37Rv	0.06	57.0	[95]
Ohmyungsamycin B					0.12	117.0	
Munumbicin B	*Streptomyces* sp. NRRL 30562	AU	Plant-associated (*Kennedia nigriscans*)	Mtb MDR	7.88	10.0	[96]
				Mtb H37Rv	36.23	46.0	
Munumbicin C				Mtb MDR	96.26	> 125	
				Mtb H37Rv	115.52	> 150	
Atratumycin	*Streptomyces atratus* SCSIO ZH16	CN	Marine sediment	Mtb H37Ra	3.8		[97]
				Mtb H37Rv	14.6		
Nosiheptide	*Streptomyces* sp. OPMA 1245	JP	Marine sediment	*M. bovis* BCG	0.01	0.012	[98]
				*M. avium *JCM15430	0.02	0.024	
				*M. intracellulare* JCM6384	0.02	0.024	
				*M. smegmatis *M341	5.11	6.25	
Griseoviridin				*M. bovis *BCG	13.09	6.25	
				*M. avium *JCM15430	3.27	1.56	
				*M. intracellulare* JCM6384	3.27	1.56	
				*M. smegmatis *M341	209.42	> 100	
Etamycin				*M. bovis *BCG	0.89	0.78	
				*M. avium *JCM15430	0.11	0.097	
				*M. intracellulare* JCM6384	0.22	0.19	
				*M. smegmatis *M341	28.44	25.0	
Mollemycin A	*Streptomyces* sp. CMBM0244	AU	Marine sediment	*M. bovis* BCG	3.2		[99]
Wollamide A	*Streptomyces* sp. MST-115088	AU	Soil	*M. bovis* BCG	2.8		[100]
Wollamide B					*3.1*		
Ecumicin	*Nonomuraea* sp. MJM5123	NA	NA	Mtb H37Rv	0.16		[101]
				Mtb H37Rv	0.12		
				Mtb	0.31		
Lassomycin	*Lentzea kentuckyensis*	US	Soil	Mtb H37Rv	0.41–0.83	0.78–1.56	[102]
				*M. avium* sub sp. Paratuberculosis	0.07–0.13	0.125–0.25	
Svetamycin A	*Streptomyces* sp. DSM 14386	DE	NA	Mtb H37Rv	103.96	65.6	[103]
				*M. smegmatis*	50.71	32.0	
Svetamycin C				Mtb H37Rv	51.93	54.0	
				*M. smegmatis*	*12.14*	8.0	
Svetamycin G				*M. smegmatis*	2.84	2.0	
Pyridomycin	*Dactylosporangium fulvum*	JP	Soil	Mtb H37Rv	0.72	0.39	[104]
				*M. bovis* BCG	0.72	0.39	
				*M. smegmatis*	1.44	0.78	
				*M. marinum*	5.79	3.13	
				*M. abcessus*	11.56	6.25	
				*M. bolletii*	11.56	6.25	
				*M. massiliense*	11.56	6.25	
				*M. avium*	23.12	12.5	
Cyclic peptide 9	*Streptomyces* sp. MS110128	CN	Marine sediment	*M. bovis *BCG	16.84	12.5	[105]
Cyclic peptide 10					16.48	12.5	
Cyclic peptide 11					1.68	1.25	
Cyclic peptide 12					1.65	1.25	
Taeanamide A	*Streptomyces* sp. AMD43	KR	Soil	Mtb mc^2^ 6230	27.0		[106]
Taeanamide B					63.0		
Thiolopyrrolone A	*Streptomyces* sp. BTBU20218885	CN	Soil	*M. bovis *BCG	16.11	10.0	[107]
				Mtb H37Rv	0.50	0.3125	
Thiolutin				*M. bovis *BCG	43.80	10.0	
				Mtb H37Rv	2.74	0.625	

NA: not available

^a^Two-letter country codes defined in ISO 3166–1.

**Table 2:** Antimycobacterial Polyketides Isolated from Actinobacteria

Metabolite	Actinobacteria specie		Country^a^	Source		Microorganism-test	MIC (μM)	MIC (μg/mL)	Reference	
Niphimycin C	*Streptomyces* sp. IMB7-145	CN		Marine sediment	Mtb H37Rv		3.50	4.0		[160]
Niphimycin Iα							3.50	4.0		
Mimosamycin	*Streptomyces lavendulae*		NA	NA		*Mycobacterium* sp. 607	107.19	25.0	[161]	
						*M. avium*	107.19	25.0		
						Mtb H37Rv	13.40	3.125		
						Mtb Matsudo	6.69	1.56		
						Mtb SM-R	6.69	1.56		
						*M. bovis* 10	26.80	6.25		
						*M. bovis* BCG	13.40	3.125		
Lincolnenin A	*Streptomyces lincolnensis* ACM-4234		US	NA		Mtb H37Ra	0.9		[162]	
Lincolnenin B							30.0			
Lincolnenin C							16.2			
Dynactin	*Streptomyces puniceus* AS13		IN	Soil		Mtb H37Rv	1.31	1.0	[163]	
Aranciamycin I	*Streptomyces* sp. CMB-M0150		AU	Marine sediment		*M. bovis* BCG	10		[164]	
Aranciamycin J							10			
Aranciamycin A							30			
Aranciamycin							30			
Azalomycin B/elaiophylin	*Streptomyces* sp. BCC71188		TH	Soil		Mtb H37Ra	0.76	0.78	[165]	
11,11'-O–dimethylelaiophylin							2.97	3.13		
Efomycin G							11.87	12.0		
Boromycin	*Streptomyces antibioticus*		CI	Soil		Mtb H37Rv	0.08		[166]	
						*M. bovis* BCG	0.2			
Lobophorin I	*Streptomyces* sp. 1053U.I.1a.3b		PH	Animal-associated (*Lienardia totopotens*)		Mtb H37Ra	2.6		[167]	
Lobophorin F							7.8			
Lobophorin B							1.3			
Lobophorin C							1.4			
Lobophorin G	*Streptomyces* sp. MS100061		CN	Marine sediment		*M. bovis* BCG	1.3	1.56	[168]	
						Mtb H37Rv	26.68	32.0		
Lobophorin A						*M. bovis* BCG	1.35	1.56		
						Mtb H37Rv	27.65	32.0		
Lobophorin B						*M. bovis* BCG	0.65	0.78		
						Mtb H37Rv	13.47	16.0		
Steffimycin B	*Streptomyces scabrisporus*		MX	Plant-associated (*Amphipterygium adstringen*)		Mtb H37Rv	13.25	7.8	[169]	
						Mtb H37Ra	0.01	0.006		
						Mtb MDR	6.63	3.9		
Steffimycin	*Streptomyces* sp. OPMA02852		US	Soil		*M. bovis* BCG	1.36	0.78	[170]	
						*M. avium*	2.72	1.56		
						*M. intracellulare*	0.68	0.39		
						*M. smegmatis*	2.72	1.56		
10-dihydrosteffimycin						*M. bovis* BCG	21.68	12.5		
						*M. avium *	5.43	3.13		
						*M. intracellulare*	1.36	0.78		
						*M. smegmatis*	5.45	3.14		
8-demethoxysteffimycin						*M. bovis *BCG	5.75	3.13		
						*M. avium*	11.48	6.25		
						*M. intracellulare*	0.72	0.39		
						*M. smegmatis*	5.75	3.13		
Steffimycin C	*Streptomyces* sp. BCC27095		TH	Soil		Mtb H37Ra	0.32		[171]	
10-Dihydrosteffimycin B							0.66			
Steffimycin B							0.0052			
7-deoxysteffimycinone							3.92			
Treponemycin	*Streptomyces* sp. MS-6–6		SA	Soil		Mtb ATCC 25177	8.52	4.17	[172]	
Chrysomycin A	*Streptomyces *sp. OA161		IN	Soil		Mtb H37Rv	6.15	3.125	[173]	
Urdamycinone E	*Streptomyces* sp. BCC45596		TH	Animal-associated (*Xestospongia sp.*)		Mtb H37Ra	5.88	3.13	[174]	
Urdamycinone G							24.31	12.5		
Dehydroxyaquayamycin							14.39	6.25		
Urdamycin E							14.03	12.5		
Kimidinomycin	*Streptomyces* sp. KKTA-0263		NA	NA		*M. bovis* BCG	23.66	25.0	[175]	
						*M. avium*	11.83	12.5		
						*M. intracellulare*	0.74	0.78		
						*M. smegmatis*	11.83	12.5		
Diazaquinomycin A	*Micromonospora maritima* B026		US	Lake sediment		Mtb H37Rv	0.28	0.10–0.72	[176]	
						MDR Mtb strains	0.17	0.06- 0.27		
						*M. abscessus*	21.16	> 7.5		
						*M. chelonae*	21.16	> 7.5		
						*M. marinum*	21.16	> 7.5		
						*M. kansasii*	21.16	> 7.5		
						*M. avium*	10.86	3.85		
						*M. smegmatis*	12.87	4.56		
						*M. bovis*	0.34	0.12		
Diazaquinomycin J						Mtb H37Rv	0.18	0.07		
Diazaquinomycin H						Mtb H37Rv	0.10	0.04		
Methyl aeruginoate	*Streptomyces *sp. TBRC7642		TH	Plant-associated (*Epipremnum aureum*)		Mtb H37Ra	105.93	25.0	[177]	
(R)-desferri-ferrithiocinmethylester							139.51	50.0		
Desferri-ferrithiocin-4-hydroxyphenethylester							34.91	12.5		
Furaquinocin D							129.40	50.0		
Murayaquinone							9.65	3.13		
Efomycin G	*Streptomyces* sp. BCC72023		TH	Plant-associated (*Oriza sativa*)		Mtb H37Ra	11.87	12.0	[178]	
Oxohygrolidine							86.99	50.0		
29-O-Methylabierixin							67.70	50.0		
Napyradiomycin B7a	*Streptomyces *sp. CA-271078		ST	Animal-associated (ascidian)		Mtb H37Ra	24.13	12.0–24.0	[179]	
Napyradiomycin SC							46.57	24.0–48.0		
Napiradiomycin D1							50.06	24.0–48.0		
3-chloro-6,8-dihydroxy-8-α-lapachone							38.87	12.0–24.0		
Napyradiomycin B6							24.09	12.0–24.0		
Napyradiomycin A2a							96.74	48.0–96.0		
Napyradiomycin A2b							96.74	48.0–96.0		
Napyradiomycin B4							22.48	12.0–24.0		
Napyradiomycin B2							50.31	24.0–48.0		
Napiradiomycin B5							45.97	24.0–48.0		
Nibomycin	*Streptomyces* sp. MS44		JP	Marine sediment		Mtb H37Rv	14.08	4.2	[180]	
						Mtb T1413	21.12	6.3		
						Mtb T1538	17.43	5.2		
Frenolicin A	*Streptomyces* sp. TBRC17107		TH	Animal-associated (*Cossus chloratus*)		Mtb H37Ra	72.19	25.0	[181]	
Frenolicin G							34.40	25.0		
Desertomycin G	*Streptomyces althioticus* MSM3		ES	Plant-associated (green algae)		Mtb H37Rv	13.28	16.0	[182]	
						Mtb MDR-1	13.28	16.0		
						Mtb MDR-2	13.28	16.0		
Desertomycin A	*Streptomyces flavofungini* TRM90047		CN	Soil		Mtb	20.96	25.0	[183]	
Desertomycin 44–1							20.98	25.0		
Desertomycin 44–2							48.56	50.0		
Panosialin A	*Streptomyces* sp. AN1761		KR	Soil		Mtb H37Rv	532.67	256.0	[184]	
Panosialin B							532.67	256.0		
Panosialin wA							319.52	128.0		
Panosialin wB							319.52	128.0		
Gwanacoside A	*Streptomyces* sp. GA02		KR	Soil		Mtb mc2 6230	28.02	15.0	[185]	
Phitsanoside B	*Streptomyces *sp. TBRC 11511		TH	Soil		Mtb	93.25	25.0	[186]	
Proximycin B	*Verrucosispora* sp. MS100047		CN	Marine sediment		Mtb H37Rv	60.47	25.0	[187]	
						*M. bovis* BCG	15.12	6.25		
Dumulmycin	*Streptomyces* sp. DM28		KR	Marine sediment		Mtb mc^2^ 6230	27.1		[188]	
Hydroxycapsimycin	*Streptomyces* sp. KKMA-0239		JP	Marine sediment		*M. avium*	92.54	> 50	[189]	
						KOmav5	92.54	> 50		
						KOmav10	92.54	> 50		
						*M. intracellulare*	92.54	50.0		
Brokamycin						*M. avium*	80.62	> 50		
						KOmav5	80.62	> 50		
						KOmav10	80.62	50.0		
						*M. intracellulare*	20.15	12.5		
Ikarugamycin						*M. avium*	52.24	25.0		
						KOmav5	104.47	> 50		
						KOmav10	104.47	50.0		
						*M. intracellulare*	52.24	25.0		
Platensimycin	*Streptomyces platensis*		NA	NA		*M. smegmatis*	31.71	14.0	[190]	
						Mtb H37Rv	27.18	12.0		
						Mtb CDC1551	27.18	12.0		
						*M. bovis* BCG	289.92	> 128		

NA: not available

^a^Two-letter country codes defined in ISO 3166–1

**Table 3:** Antimycobacterial metabolites from Actinobacteria: Nucleotides, aminoglycosides, and miscellaneous compounds

Metabolite	Actinobacteria specie	Country^a^	Source	Microorganism-test	MIC (μM)	MIC (μg/mL)	Reference
Mavintramycin A	*Streptomyces* sp. OPMA40551	JP	Marine sediment	*M. avium* JCM15430	1.62	0.78	[239]
				*M. intracellulare* JCM6384	0.81	0.39	
				*M. smegmatis* mc^2^ 155	6.50	3.12	
				*M. bovis* BCG	3.25	1.56	
Mavintramycin B				*M. avium* JCM15430	12.30	6.25	
				*M. intracellulare* JCM6384	6.14	3.12	
				*M. smegmatis* mc^2^ 155	24.59	12.5	
				*M. bovis* BCG	12.30	6.25	
Mavintramycin C				*M. avium* JCM15430	104.11	> 50	
				*M. intracellulare* JCM6384	52.06	25.0	
				*M. smegmatis* mc^2^ 155	104.11	> 50	
				*M. bovis* BCG	13.01	6.25	
Mavintramycin D				*M. avium* JCM15430	51.84	25.0	
				*M. intracellulare* JCM6384	12.96	6.25	
				*M. smegmatis* mc^2^ 155	51.84	25.0	
				*M. bovis* BCG	12.96	6.25	
Mavintramycin E				*M. avium* JCM15430	21.28	12.5	
				*M. intracellulare* JCM6384	21.28	12.5	
				*M. smegmatis* mc^2^ 155	85.14	50.0	
				*M. bovis* BCG	10.64	6.25	
Mavintramycin F				*M. avium* JCM15430	6.31	3.12	
				*M. intracellulare* JCM6384	1.58	0.78	
				*M. smegmatis* mc^2^ 155	6.31	3.12	
				*M. bovis* BCG	3.16	1.56	
Mavintramycin G				*M. avium* JCM15430	6.35	3.12	
				*M. intracellulare* JCM6384	1.59	0.78	
				*M. smegmatis* mc^2^ 155	25.45	12.5	
				*M. bovis* BCG	0.79	0.39	
Amicetin				*M. avium* JCM15430	5.04	3.12	
				*M. intracellulare* JCM6384	5.04	3.12	
				*M. smegmatis* mc^2^ 155	2.52	1.56	
				*M. bovis* BCG	0.63	0.39	
Plicacetin				*M. avium* JCM15430	6.03	3.12	
				*M. intracellulare* JCM6384	6.03	3.12	
				*M. smegmatis* mc^2^ 155	6.03	3.12	
				*M. bovis* BCG	1.51	0.78	
Caprazamycin B	*Streptomyces* sp. MK730-62F2	JP	NA	Mtb H37Rv	2.73	3.13	[240]
				*M. bovis*	2.73	3.13	
				Drug susceptible Mtb	5.45	6.25–12.5	
				MDR Mtb	5.45	6.25–12.5	
				*M. avium*	5.45	6.25–50.0	
				*M. intracellulare*	1.36	1.56–25.0	
Streptothricin E	*Streptomyces* sp. I08A 1776	CN	Soil	Mtb H37Rv	1.59	1.0	[241]
				Mtb 164	0.79	0.5	
				Mtb 926	0.40	0.25	
Streptcytosine A	*Streptomyces* sp. TPU1236A	JP	Marine sediment	*M. smegmatis*	50.74	32.0	[242]
Plicacetin					61.82	32.0	
Bamicetin					26.46	16.0	
Carbazomycin D					87.61	25.0	
Pimprinine					126.12	25.0	
Kandenol A	*Streptomyces* sp. HKI0595	CN	Plant-associated (*Kandelia candel*)	*M. vaccae* IMET 10670	49.53	12.5	[243]
Kandenol B					46.58	12.5	
Kandenol C					43.96	12.5	
Kandenol D					93.16	25.0	
Kandenol E					49.93	12.5	
(2S,2"S)-6-lavandulyl-7,4′-dimethoxy-5,2′-dihydroxylflavanone	*Streptomyces* sp. G248	VN	Animal-associated (*Halichondria panicea*)	Mtb H37Rv	106.15	48.0	[244]
(2S,2"S)-6-lavandulyl-7-methoxy-5,2′,4′-trihydroxyflavanone					13.69	6.0	
6-prenyl-4′-methoxy-5,7-dihydroxyflavanone					31.32	11.1	
2,4-di-tert-butylphenol	*Streptomyces bacillaris *ANS2	IN	Soil	Mtb MDR	242.37	100.0–50.0	[245]
				Mtb H37Rv	48.47	10.0–50.0	

NA: not available

^a^Two-letter country codes defined in ISO 3166–1

#### Peptide antibiotics

The non-ribosomal peptide synthesis (NRPS) pathway is mediated by large multifunctional enzymes and is characterized by the production of non-ribosomal peptides (NRP), which are not encoded by genes and are not restricted to the 20 conventional amino acids. Non-ribosomal peptide synthetases (NRPS) use proteinogenic and non-proteinogenic amino acids as building blocks for the peptide chain. As a result, the products generated by this pathway present a great structural diversity and a broad spectrum of biological activities, making them valuable for application in agriculture and medicine [78–80]. Some common characteristics among NRP include their highly specific structure, which ensures bioactivity through a precise orientation, essential to interact with a molecular target; in addition, macrocyclization is an important characteristic, in which distant parts of the linear peptide precursor are covalently linked to each other [81]. Table 1 presents NRP isolated from different Actinomycetota species, along with their respective MIC values against mycobacterial strains.

*Actinomycins.* Actinomycins are chromogenic cyclic peptides, first discovered in 1940 [108]. These substances stand out for being antibiotics and anticancer agents, isolated from several species of *Streptomyces*, of which, to date, about 30 natural and synthetic analogues have been discovered [109, 110]. These actinomycins are composed of a chromophore group and two pentapeptide chains, whose amino acid composition varies (Fig. 3).

Our analysis revealed that actinomycins exhibiting antitubercular activity were isolated from five distinct *Streptomyces* species, including *S. griseoruber, S. smyrnaeus, S. avermitilis*, and two unclassified *Streptomyces* species. Praveen and Tripathi [82] evaluated the actinomycin D activity against Mtb H37Rv, reporting a MIC value of 0.62 µM. Qureshi et al. [83] isolated both actinomycin D and actinomycin X_2_, which exhibited MIC values of 2.08 µM and 1.43 µM, respectively, against Mtb H37Rv. Similarly, Chen et al. [84] reported the isolation of actinomycin D, actinomycin X_2_, and actinomycin X0β. These compounds displayed MIC of 6.37 µM, 0.79 µM, and 6.29 µM, respectively, against Mtb H37Rv. In another study, Shah et al. [85] isolated actinomycin D, actinomycin X_2_, and actinomycin C3. These compounds demonstrated particularly potent activity against Mtb H37Rv, with MIC of 0.05 µM, 0.03 µM, and 0.03 µM, respectively. Rakhmawatie et al. [86] also reported the isolation of actinomycin D, which exhibited an MIC of 0.62 µM.

Structurally, actinomycins X_2_ and X0β are closely related to actinomycin D, with variation arising from the substitution of the proline residue in the polypeptide ring. Replacement by 4-trans-hydroxyproline or 4-oxo-proline yields actinomycin X0β and X2, respectively [111] (Fig. 3). In contrast, actinomycin C3 contains modifications in the D-valine residues of the polypeptide ring, which are replaced by D-allo-isoleucine [111] (Fig. 3). Among these compounds, actinomycin D is the most studied analog and continues to be widely used in the treatment of various types of cancer, especially in pediatric oncology and has been gaining prominence for its antimicrobial potential [112].

From a biochemical point of view, actinomycin D's mechanism of action is based on its ability to intercalate between GC base pairs, preventing the progression of RNA polymerase and, blocking gene transcription [113, 114]. Despite their high bioactive potential, the toxicity associated with actinomycins still represents a major obstacle to their wider clinical use [115]. However, given the global crisis of antimicrobial resistance and the urgent need for new anti-TB therapies, the re-evaluation of classic molecules, such as actinomycins, has emerged as a viable strategy. The rational use of these molecules, combined with structural modification approaches, encapsulation or combination with other agents, can reduce their side effects and broaden their therapeutic applicability.

*Echinomycin.* Echinomycin is a cyclic depsipeptide antibiotic known for its extensive activities against bacteria and tumor cells (Fig. 3). This compound belongs to the quinoxaline family and originates from different species of *Streptomyces*, Echinomycin promotes DNA damage, cell apoptosis and inhibition of bacterial RNA synthesis [89]. This substance was the first DNA bisintercalator identified, having the ability to reversibly bind to the double helix in a sequence-independent fashion, inserting one or more aromatic ring groups between adjacent base pairs. Thus, echinomycin bound to DNA inhibits transcription in bacteria, chromatin condensation and DNA replication in eukaryotic organisms, leading to cell cycle arrest [116].

The study conducted by Chen et al. [89] observed that echinomycin obtained exhibited a MIC of 0.45 μM against Mtb H37Rv. However, according to Gade et al. [117], despite its potent antimicrobial activity, echinomycin is not used clinically due to solubility and toxicity concerns. The study of Foster et al. [118] investigated the toxicity of echinomycin in mice and Beagle dogs through intravenous injections administered over five consecutive days, and the primary toxic effects were observed in the gastrointestinal, hepatic, and lymphoreticular systems.

*Cyclomarins.* Cyclomarins A and C are heptapeptide cyclopeptides biosynthesized via the NRPS, sharing a similar central structure but presenting crucial differences in their side chains (Fig. 3). Cyclomarin A demonstrated high activity against the virulent strain of Mtb, with a MIC of 0.48 µM [87] while cyclomarin C was capable of inhibiting Mtb H37Ra with a MIC of 0.10 µM [88].

The main distinction between these two compounds lies in the chemical modification of the hydroxylated tryptophan residue. Cyclomarin A contains an epoxide group on the side of this residue, specifically an N-(1,1-dimethyl-2,3-epoxypropyl) substitution [119, 120]. This structural difference has direct implications for the bioactivity of the compounds. Studies have shown that the presence of the epoxide group in cyclomarin A is essential for its potent activity against Mtb, since this functional group is involved in the interaction with the target protein caseinolytic protease C1 (ClpC1). In contrast, cyclomarin C, which lacks this modification, has considerably reduced antimicrobial activity, showing that small structural changes can significantly impact the pharmacological efficacy of the molecule [119].

Currently, the information available on the toxicological profile of cyclomarins is still scarce. The presence of the epoxide group in the structure of cyclomarin A, although essential for its bioactivity, represents a highly reactive functional structure that can interact non-specifically with host macromolecules, raising concerns about toxicity [119, 121]. The possibility of side effects related to cross-inhibition of human proteases or other homologous structural chaperones represents a significant limitation for the clinical use of these compounds, if a satisfactorily high therapeutic index is lacking [114, 122–124].

*Rufomycins and ilamycins*. Rufomycins and ilamycins are cyclic heptapeptides that feature an isoprenyl group attached to the nitrogen of the tryptophan ring [125] (Fig. 3). These compounds have been increasingly recognized for their potential in the treatment of TB, particularly for their inhibitory action on ClpC1, a validated and essential target for Mtb viability [126].

In the study conducted by Zhou et al. [90] eight new rufomycins compounds were evaluated for their antimycobacterial activity against a virulent strain of Mtb H37Rv. The results revealed a broad range of potencies, with MIC values ranging from 0.030 μM to greater than 10 μM. The presence of an epoxide ring in the prenyl group of tryptophan was strongly correlated with increased antimicrobial activity, while the absence of functional groups such as N-methylleucine (N-MeLeu) and m-nitro-tyrosine (m-NO₂-Tyr) resulted in the compounds losing their efficacy.

Sun et al. [91] isolated twelve ilamycins (G to R, also referred to as rufomycins), and evaluated their activity against Mtb H37Rv, yielding MIC of 0.0096–9.6 μM (Table 1). It was observed that structural modification involving reduction and cyclization at position C-33 significantly enhances the biological activity of these compounds. In contrast, oxidation at C-15 did not substantially affect activity, while oxidation at C-32 resulted in a loss of efficacy. Conversely, in another analysis, oxidation at C-15 compromised activity, whereas oxidation at C-32 contributed positively. Furthermore, the presence of a nitro group at C-43 plays a crucial role in maintaining or enhancing the observed antitubercular activity.

Although the bioactivity data is significant, authors also reported concerns about the toxicity of these compounds. Rufomycins, in particular, showed relevant cellular toxicity in murine macrophage models, with relatively low selectivity indices for some analogues, which may limit their direct clinical application without additional structural modifications [90]. These findings indicate that, although potent, these compounds may interfere with conserved cellular pathways in host cells, which calls for further investigation into their safety. Thus, the studies involving rufomycins and ilamycins reinforce the high potential of these cyclopeptides as ClpC1 inhibitors, representing a promising new class of anti-TB agents.

*Tuberactinomycins*. Tuberactinomycins are a family of cyclic peptide antibiotics, including viomycin and capreomycin, that exhibit potent activity against Mtb*,* particularly MDR and XDR strains. Structurally, they feature a unique hexapeptide core with non-proteinogenic amino acids and a guanidine group critical for ribosomal targeting [127] (Fig. 4). Their mechanism involves binding to the 30S and 50S ribosomal subunits at the interface, specifically disrupting tRNA accommodation during translocation (A- to P-site movement) and inhibiting protein synthesis.

Tuberactinomycins have historically been employed as second-line therapeutic agents in the treatment of MDR-TB [127]. However, despite their efficacy, clinical use is limited by nephrotoxicity and ototoxicity, though they remain WHO-recommended for MDR-TB when safer options (e.g., bedaquiline) are unavailable.

Viomycin was the first member of the class to be isolated and was later identified as chemically identical to tuberactinomycin B. Capreomycin, isolated from *Streptomyces capreolus*, was initially described as a mixture of four distinct components (capreomycins IA, IB, IIA, and IIB), which were subsequently differentiated based on structural substituents. Despite this chemical complexity, capreomycin has been clinically used as a combined formulation of all four components.

In the study by Rokuro et al. [92] tuberactinomycins A and B was isolated from the soil actinobacteria *Streptomyces griseoverticillatus* and inhibited Mtb ATCC 607 with MIC of 17.81 μM and 4.67 μM, respectively. Although current clinical guidelines restrict their use due to toxicity concerns, their retained efficacy against resistant strains suggests potential for revitalization. Prior research indicates that modulating culture conditions [128] or engineering BGC could yield novel analogs with improved pharmacological profiles. Semi-synthetic modifications of these ciclopeptides may further expand the therapeutic utility. Beyond TB, repurposed tuberactinomycins could address other priority pathogens, as evidenced by viomycin’s activity against other priority pathogens, as vancomycin-resistant *Enterococcus* (VRE) and methicillin-resistant *Staphylococcus aureus* (MRSA) [129, 130].

*Hytramycins*. Hytramycin I and V are cyclohexapeptides, each bearing three unusual piperazic acid moieties, two adjacent and the third flanked by another amino acid [93, 131] (Fig. 4). Both compounds were tested against Mtb H37Rv and exhibited MIC of 17.84 μM and 9.27 μM, respectively. In addition, the MIC against nonreplicating Mtb fall into the range of some existing second-line anti-TB drugs, such as streptomycin and capreomycin, respectively. However, the IC_50_’s of hytramycin V and I against Vero cells were 24.2 and 7.7 μg/mL, resulting in rather poor selectivity indices (SI = IC_50_/ MIC) [93].

*Atrovimycin*. Atrovimycin is a cyclodepsipeptide isolated from *Streptomyces atrovirens* LQ13 and showed promising antimycobacterial activity, with an MIC value of 1.88 µM against Mtb H37Rv [94]. Structurally, atrovimycin stands out for the presence of a vicinally hydroxylated acyl cinnamic chain, considered rare and potentially relevant to its biological activity (Fig. 4).

In view of the good in vitro results, the authors moved on *to *in vivo tests in murine model of TB*.* The effects of oral administration of atrovimycin were compared with those of two reference drugs in the treatment of TB, rifampicin and ethambutol. The results revealed that atrovimycin was able to significantly reduce the pulmonary bacterial load, achieving an efficacy similar to that of ethambutol. Compared to rifampicin, considered the gold standard in the treatment of TB, atrovimycin showed slightly less activity, although still significant. Importantly, while rifampicin promoted a more marked reduction in bacterial load, atrovimycin demonstrated a superior safety profile, once the treated mice showed no obvious signs of clinical or behavioral toxicity, and histological examinations indicated no relevant liver or kidney damage [94]. These data suggest that atrovimycin combines significant anti-TB activity with low toxic potential, making it a promising candidate for future stages of pre-clinical development.

*Ohmyungsamycins*. Ohmyungsamycin A and ohmyungsamycin B are characterized as cyclic peptides with cytotoxic and antimicrobial activities [132] (Fig. 4). These compounds were tested against Mtb H37Rv and exhibited MIC values of 0.06 μM and 0.12 μM, respectively. In mice infected with Mtb, the compounds were able to activate the autophagy pathway mediated by AMP-activated protein kinase, promoting the intracellular elimination of the bacillus by macrophages. This mechanism is of great relevance, since the activation of autophagy represents an effective cellular strategy in containing chronic infections, such as TB [95]. At the same time, a negative modulation of the exacerbated inflammatory response was observed, with a significant reduction of pro-inflammatory cytokines such as TNF-α and IL-6 in lung tissues.

The dual action of these compounds, as antimicrobial agents and immunomodulators, extends their therapeutic value, especially in the context of persistent infections and resistant forms of TB, where the immune system's ability to control the pathogen is limited. Moreover, the compounds showed no significant toxicity for *Drosophila melanogaster* flies, which reinforces their preliminary pharmacological safety and viability as candidates for clinical development.

*Munumbicins*. Munumbicins belong to the group of polycyclic aromatic antibiotics with antimicrobial, antimalarial and antitumor properties. Structurally, munumbicins are classified as cyclic depsipeptides with variations in aromatic substituents and side chains, which confer different degrees of polarity, stability and biological activity [96].

Castillo et al. [96] isolated munumbicin B and munumbicin C (Fig. 4) and tested against Mtb H37Rv, yielding MIC values of 35.43 μM and 115.52 μM, and against multidrug-resistant MDR Mtb, with MIC of 7.88 μM and 98.46 μM, respectively. The authors state that although munumbicins have therapeutic potential and activity against cancer cells, their clinical advancement depends on additional studies of cellular and clinical toxicity, since they demonstrate toxic effects in high concentrations on normal human cells. Therefore, chemical modification of these compounds, aiming to reduce toxicity and increase efficacy, may be an essential strategy to enable their use as antimalarial, anticancer or anti-infectious agents [96].

*Atratumycin*. Atratumycin is a cyclic decapeptide featuring a unique 3-(2-methylphenyl)-2(E)-propenoic acid moiety appended to the amino acid chain (Fig. 4). Atratumycin was tested against Mtb H37Ra and H37Rv and exhibited a MIC of 3.8 and 14.6 μM, respectively [97]. Although the MIC value is slightly higher when compared to other natural products such as cyclomarins or ilamycins, the therapeutic relevance of atratumycin is reinforced by its favorable toxicity profile, which is a critical attribute in the early stages of drug development.

Regarding cytotoxicity, atratumycin was tested against a panel of human cancer cell lineages as well as non-cancerous human lineages. The compound exhibited no significant toxicity across all tested cell lines even at high concentrations (> 50 μM), suggesting low off-target cytotoxicity and high therapeutic selectivity. Based on these findings, the authors proposed atratumycin as promising candidate for the development of novel anti-TB agents, especially due to its balanced profile of antimicrobial efficacy and cellular safety. Considering their structural features, the cyclic peptide backbone, combined with the unique aromatic moiety, offers a structurally versatile scaffold that may be further optimized through synthetic modification to enhance potency and selectivity.

*Nosiheptide, griseoviridin and etamycin*. Nosiheptide, also known as multiomycin, is a thiopeptic antibiotic isolated in 1970 [133] (Fig. 4). Its mechanism of action is based on the inhibition of bacterial protein synthesis by blocking the elongation factors EF-Tu and EF-G, which are essential for the ribosomal translation process [134]. Griseoviridin and etamycin (viridogrisein) (Fig. 4) are natural antibiotics of the streptogramin class, belonging to distinct subclasses that act synergistically, enhancing antibacterial activity [135].

In the study conducted by Hosoda et al. [98], three compounds were isolated from *Streptomyces* sp. OPMA1245, obtained from marine sediments in Japan, and were tested against *M. bovis*, *M. avium*, *M. intracellulare*, and *M. smegmatis*. The study show that the three substances have remarkable inhibitory activity. Nosiheptide exhibited MIC values of 0.01 μM, 0.02 μM, 0.02 μM, and 5.11 μM, respectively. Griseoviridin showed MIC of 13.09 μM, 3.27 μM, 3.27 μM, and 209.42 μM, respectively. Finally, etamycin demonstrated MIC values of 0.89 μM, 0.11 μM, 0.22 μM, and 28.44 μM, respectively.

These antibiotics act by inhibiting bacterial protein synthesis, a mechanism of action that may be particularly effective against mycobacteria of the *M. avium* complex, considering the peculiar structure of their cell wall and their recognized resistance to multiple drugs. Considering the limitations of currently available treatments for infections caused by this mycobacterial complex, the results obtained reinforce the relevance of these substances as promising candidates for the development of new antibiotics or complementary therapeutic strategies.

*Mollemycin.* Raju et al. [99] were the first to isolate mollemycin A, a glyco-hexadepsipeptide-polyketide (Fig. 4) with a inhibitory effect against *M. bovis* BCG, with a MIC of 3.2 μM. Notably, it showed significantly reduced cytotoxicity against mammalian cell lines [136]. Beyond antimycobacterial activity, mollemycin A also exhibits strong antimalarial activity against both sensitive and multidrug-resistant *Plasmodium falciparum* strains, combined with low toxicity in mammalian cells.

*Wollamides.* Wollamides are cyclic hexapeptides that have gained attention due to their promising in vitro antimycobacterial activity, particularly against *M. bovis* BCG (Fig. 5). Moreover, these substances exhibit no significant cytotoxicity in a mammalian cell panel, making them attractive candidates for the development of antimicrobial therapies, especially in the fight against TB [137].

Khalil et al. [100] isolated wollamide A and B, which demonstrated potential activity against *M. bovis* BCG. Wollamide A is a cyclic macro-lactam composed of a sequence of amino acids linked by peptide bonds, including units of glutamic acid and 2-amino-3-hydroxy-5-phenylpentanoic acid, with a lactam ring in its structure. On the other hand, amino acid composition in wollamide B reflects over their biological activity, leading to a slight reduction of antimycobacterial response, compared to wollamide A. The compounds exhibited MIC values of 2.8 μM and 3.1 μM, respectively.

Rollo et al. [138] investigated the interaction of these substances with first- and second-line TB antibiotics. As a result, they showed that wolamide B1 exhibited significant antimycobacterial activity and, when combined with these antibiotics, enhanced their efficacy against Mtb, including MDR and XDR strains. Furthermore, the compound did not interfere with the action of first-line antibiotics, suggesting it as a promising alternative for combination therapies in the fight against TB, particularly against resistant strains.

*Ecumicin.* Ecumicin (Fig. 5) consists of a macrolactam ring containing multiple amino acid residues, including N-methylated units and non-proteinogenic amino acids, as well as ester functional groups and hydrophobic side chains that enhance its interaction with cellular targets and contribute to its conformational stability and resistance to enzymatic degradation [139].

Ecumicin exhibits high potency against Mtb, acting through a mechanism distinct from that of traditional antibiotics. Studies have shown that its primary target is the GroEL/ES chaperonin complex, which is essential for proper protein folding, leading to effective inhibition of bacterial growth [140]. Of importance, an ecumicin analogue demonstrated in vitro intracellular activity in macrophage model of mycobacterial infections and maintained its activity for 28 days in time-kill tests, under different growth conditions [141]. Additionally, ecumicin displays a favorable toxicological profile, with low cytotoxicity in human cells, reinforcing its potential as a candidate for the development of new therapeutic agents against TB, including multidrug-resistant strains [139, 140]. The study conducted by Gao et al. [101] observed a MIC of 0.16 μM. This substance acts by inhibiting the ClpC1/ClpP1/ClpP2 proteolytic complex, which is essential for the degradation of damaged proteins in Mtb. It binds to the N-terminal domain of the ClpC1 protein and blocks its function in a concentration-dependent manner. In addition, it strongly stimulates the ATPase activity of ClpC1, accelerating ATP hydrolysis and disrupting the bacterium's energy balance [142].

Complementing these findings, Hawkins et al. [143] reported the first total synthesis of ecumicin, using an efficient convergent route to construct the highly functionalized peptide core of the molecule. The synthetic ecumicin was subjected to antimicrobial assays and retained significant activity against Mtb H37Rv, with a MIC₉₀ of 312 nM. This result confirms that the potent activity observed in natural products could be achieved by inspiring chemical synthesis.

*Lassomycin.* Belonging to the class of lasso peptides, it stands out as a ribosomally synthesized and post-translationally modified natural product (RiPP) [144] (Fig. 5), featuring a unique structure resembling a sliding knot that provides high stability and various pharmacological activities, especially a significant antitubercular activity [145]. The selectivity of its action occurs because lassomycin targets the ClpC1 ATPase, a vital enzyme in mycobacteria that plays an essential role in protein degradation in conjunction with the proteolytic complex ClpP1P2 [146].

Gavrish et al. [102] isolated lassomycin from *Lentzea kentuckyensis* that showed a MIC of 0.41 μM against Mtb H37Rv. Additionally, lassomycin demonstrated resistance to serum proteases and did not significantly bind to plasma proteins, indicating favorable pharmacokinetic properties and metabolic stability. The substance also did not cause hemolysis and exhibited low cytotoxicity (IC_50_ ~ 350 μg/mL) in human cells. Moreover, lassomycin exhibited excellent bactericidal activity against Mtb in the exponential growth phase, with potency comparable to rifampicin. In Mtb cells in the stationary phase, lassomycin was more effective than rifampicin, without the presence of surviving persistent cells. Its efficacy against dormant forms and resistant strains, combined with its innovative mechanism of action, positions it as a promising candidate for the development of new treatments for the disease.

*Svetamycins.* Svetamycins are cyclic depsipeptides, a class of natural metabolites characterized by the simultaneous presence of peptide and ester bonds within a macrocyclic framework. Chemically, these molecules incorporate non-proteinogenic amino acid residues and unusual units such as piperazic acid and halogenated aromatic groups, which contribute to their structural complexity and biological activity. Their macrocyclic architecture and lipophilic side chains suggest favorable interactions with cellular membranes or protein targets essential for microbial viability [103].

In the study conducted by Dardic et al. [103], svetamycins A, C, and G (Fig. 5) were evaluated for their antimycobacterial activity against Mtb H37Rv and *M. smegmatis*. Against *M. smegmatis*, svetamycin G demonstrated the highest potency (2.84 μM), while svetamycin C (12.14 μM) was approximately four times more active than svetamycin A (50.71 μM). The authors concluded that methylation at the δ-position of the piperazic acid unit enhances antimycobacterial activity and that replacing a chlorine atom with bromine, as seen in svetamycin G, also has a beneficial effect on antimicrobial potency.

These SAR data highlight the potential of the svetamycin scaffold as a platform for chemical optimization. The high activity of svetamycin G suggests that modifications in halogenation patterns may be an effective strategy to enhance antimycobacterial efficacy [147].

*Pyridomycins.* Pyridomycin is a cyclodepsipeptide produced by *Streptomyces pyridomyceticus* NRRL B-2517 [148]. It features a 12-membered central ring composed of two pyridyl groups, a propionic acid, and a structurally unique 2-hydroxy-3-methylpent-2-enoic acid [149]. This metabolite present a notable antimycobacterial activity, recognized for their selective action on the enoyl reductase (InhA) enzyme [150]. Their efficacy is directly related to the presence of pyridyl moieties in their structure, which play a crucial role by interacting with the reduced form of NADH cofactor binding site, thereby blocking the biosynthesis of mycolic acids, essential components for the integrity of the mycobacterial cell wall [151].

In the study by Hartkoorn et al. [104] pyridomycin (Fig. 5) demonstrated activity against all tested *Mycobacterium* species, notably Mtb H37Rv, *M. bovis* BCG, and *M. smegmatis*, with MIC values of 0.72 μM, 0.72 μM, and 1.44 μM, respectively. No effects were observed against other evaluated bacteria, leading the authors to conclude that pyridomycin acts on a specific mycobacterial target. Additionally, cytotoxicity assays revealed higher selectivity of the compound for Mtb compared to the human cell lines, reinforcing its potential as a lead compound for the development of new antitubercular drugs.

*Thiolopyrrolone A and thiolutin*. Both compounds are natural metabolites belonging to the dithiolopyrrolone class, structurally distinct compounds that exhibit a broad spectrum of antimicrobial activity [107]. Most analogs within this class possess a bicyclic pyrrolinone-dithiol core, with structural variations primarily at the N-4 and N-7 substituent positions. Thiolopyrrolone A represents the first identified member featuring a macrocyclic skeleton, structurally distinguishing it from other compounds in the class [107] (Fig. 5).

Thiolutin is a potent RNA polymerase inhibitor in both bacteria and fungi. It is characterized as a bicyclic antibiotic, defined by a disulfide bond between two ene-thiol units [152, 153]. This compound affects multiple cellular pathways, including glucose metabolism, Tor signaling, mRNA degradation, oxidative stress response, proteasome activity, and Hog/MAPK pathway signaling [154].

In the study by Song et al. [107], thiolopyrrolone A and thiolutin inhibited the virulent strain of Mtb, with MIC values of 2.74 μM and 0.5 μM, respectively. Dithiolopyrrolones are known for their broad-spectrum antibacterial effects and notable antiangiogenic activity, making them biologically promising compounds. However, their clinical application has not yet been realized due to their toxicity, which limits their therapeutic advancement [155].

*Taeanamides.* Taenamides constitute a class of nonribosomal lipopeptides. In the study conducted by Cui et al. [106] taeanamide A and taeanamide B were isolated from a *Streptomyces* sp. AMD43 (Fig. 5). Both substances exhibit a peptide structure associated with an unusual lipid chain. Taeanamide B displays a structure highly similar to that of taeanamide A, differing by the presence of an additional methoxy group, which represents a subtle yet significant structural variation between the two compounds.

When evaluated for antimycobacterial activity against Mtb mc^2^ 6230, taeanamide A and B showed MIC values of 27.0 and 63.0 μM, respectively. Subsequently, their cytotoxicities were tested against various human cancer cell lines. Taeanamide A did not demonstrate significant cytotoxic activity (IC₅₀ > 20 μM), whereas taeanamide B exhibited strong inhibition of several cancer cell lines, with IC₅₀ values ranging from 0.26 to 1.13 μM. These results highlight that the structural difference between taeanamides A and B, particularly the presence of the methoxy group in the linear form of taeanamide B, is directly associated with its higher cytotoxic activity compared to the cyclized form of taeanamide A.

Therefore, although the compounds analyzed here present antimicrobial activity classified as weak according to conventional parameters, their NRPS origin gives them great strategic value. They are highly diversifiable molecules that can be converted into promising clinical prototypes through modern techniques of medicinal chemistry, functional genomics and molecular modeling. Such perspectives reinforce the importance of the continuous prospecting of natural products originating from extreme environments that act as reservoirs of actinobacteria producing bioactive metabolites with emerging applications in antimycobacterial pharmacotherapy.

#### PKS-derived pathway antimycobacterial metabolites

The polyketide synthase (PKS) pathway is responsible for the production of secondary metabolites with high bioactive potential [156]. This pathway is capable of constructing polyketides through the sequential condensation and modification of acyl building blocks, thus generating a heterogeneous group of compounds, such as polyethers, polyenes, polyphenols, macrolides and enediynes [157]. These PKS have a specific enzymatic domain structure, in which acyl transferase, ketosynthase, and an acyl transport protein form the central structure for condensation of acyl units, being structures considered essential for PKS [158, 159]. The Table 2 presents PKS metabolites isolated from different Actinomycetota species, along with their respective MIC values against mycobacterial strains.

*Niphimycins*. Niphimycins are macrolide antibiotics characterized by the presence of an alkylguanidyl side chain and are recognized for their ability to disrupt cellular membranes [191] (Fig. 6). Hu et al. [160] isolated niphimycin C and niphimycin Iα from *Streptomyces* sp. IMB7-145. Both compounds share a macrolactone core and differ in the position of the malonyl group. In niphimycin C is esterified to a hydroxyl, whereas in niphimycin Iα, it is linked to the amino group of the guanidyl side chain. Despite this structural difference, both compounds exhibited identical MIC of 3.5 μM against Mtb H37Rv, indicating that the position of the malonyl substituent did not significantly influence their antimycobacterial activity.

*Mimosamycins*. Mimosamycin is a natural compound with antimycobacterial activity, originally isolated by Mikami et al. [161]. Subsequently, Kesteleyn and De Kimpe [192] characterized mimosamycin as the first member of a new class of natural antibiotics known as 3,5,8(2H)-isoquinolinetriones (Fig. 6). In the study by Mikami et al. [161], mimosamycin was evaluated against several mycobacterial strains with MIC values ranging from 6.69 μM to > 100 μM, depending of species tested (Table 2), identifing mimosamycin as a novel antibiotic with specific activity against mycobacteria.

*Lincolnemins*. Lincolnemycins are aromatic antibiotics characterized by a polycyclic benzophenanthrene-type system with functional groups such as hydroxyls, ketones, and, in some cases, conjugated sugars (Fig. 6) [193]. These compounds exhibit significant antimicrobial activity, particularly against Gram-positive bacteria such as MRSA and *Bacillus subtilis* [194]. Their mechanism of action is generally attributed to DNA intercalation, inhibiting processes such as replication and transcription.

In the study conducted by Mohamed et al. [162], Lincolnemins A, B, and C were evaluated for their antimycobacterial activity against Mtb H37Ra. The difference between lincolnemins A, B, and C lies in their stereochemical variations, resulting in different isomers (Fig. 6). These three compounds exhibited MIC of 0.9 μM, 30 μM, and 16.2 μM, respectively. Furthermore, this study highlighted that these compounds possess significant bactericidal activity against Gram-positive bacteria, including MRSA, suggesting their great potential in combating infections caused by resistant strains, a growing therapeutic challenge in the treatment of both hospital and community-acquired infections. Although cytotoxicity remains a challenge, the presence of multiple functional sites in lincolnemycins allows structural modification to improve their pharmacological profile. The study of their biosynthetic clusters and rational derivatization strategies make these molecules promising candidates for the development of new antibiotics and antitumor agents [193].

*Dinactin.* Dinactin (Fig. 6) is an antibiotic of the macrotetrolide family, known for its wide range of biological activities, especially its antimicrobial [195–199]. In the studies of Hussain et al. [163], dinactin inhibited a virulent strain of Mtb with an MIC of 1.31 μM. The mechanism of antimycobacterial activity of dinactin consists in damaging bacteria cell membranes, resulting in the extravasation of intracellular content [198].

*Aranciamycins*. Belonging to the anthracycline class, these compounds were described by Khalil et al. [164] as relatively rare microbial metabolites, distinguish themselves from other well-known anthracycline antibiotics, such as doxorubicin and daunorubicin by the absence of an amino group in their sugar fraction (Fig. 6). Four aranciamycins (I, J, A and aranciamycin) were evaluated for their antimycobacterial activity against *M. bovis* BCG, exhibiting MIC of 10 μM, 10 μM, 30 μM, and 30 μM, respectively [164]. The four substances share the basic structure of anthracyclines, characterized by an aromatic ring core with specific functional groups.

Based on these results, the authors concluded that the four compounds demonstrated moderate to weak antimicrobial activity. Additionally, the researchers conducted cytotoxicity tests on these compounds and observed that aranciamycin I and J exhibited in vitro cytotoxicity against human cancer cell lines. Therefore, these metabolites could serve as promising leads for developing next generation antimycobacterial agents.

*Elaiophylin and related compounds*. Elaiophylin, a macrolide composed of a 16-membered ring with symmetric glycosylation at position C2 [200, 201] (Fig. 6). Is recognized for its broad range of pharmacological activities, including antibacterial, antifungal, and immunosuppressive effects [202–204]. Elaiophylin was later identified in various *Streptomyces* species, accompanied by eighteen structurally similar analogs, among which 11,11'-O-dimethylelaiophylin and efomycin G stand out, showing variations in the functional groups at positions C-2/C-2', C-11/C-11', and C-14/C-14', while maintaining conservation in the diolide core, hemicetal fractions, and 6-deoxyfucose [205]. This substance exhibits significant antimicrobial activity against Gram-positive bacteria, including pathogenic microorganisms with drug resistance, such as MRSA and VRE. However, although the mechanism responsible for its antibacterial activity is not yet fully elucidated, it is presumed to be related to elaiophylin's ability to form selective cationic ion channels, in a stable and long-lasting manner, in lipid bilayer membranes [205].

Supong et al. [165] reported that three substances exhibited activity against Mtb H37Ra, elaiophylin, 11,11'-O-dimethylelaiophylline, and efomycin G, with MIC of 0.76 μM, 2.97 μM, and 11.87 μM, respectively. The antimicrobial activity of the analyzed compounds is intrinsically related to the presence of hydroxyl groups at positions C-11 and C-11′ in the molecular structure. These groups are essential for the formation of hemiacetals, playing a fundamental role in the interaction of the molecule with bacterial targets, determining its efficacy against bacteria. When structural modifications occur, such as by substituting the hydroxyl groups, the molecule's ability to act as an antibiotic is significantly compromised, as such alterations reduce its capacity to interact efficiently with cellular targets [206].

*Boromycin*. Boromycin (Fig. 6) is a macrolide antibiotic agent that exhibits strong action against certain viruses, Gram-positive bacteria and parasitic protozoa [166, 207–209]. This molecule contains boron in the lipophilic macrocycline lactone ring, thus becoming the first known antibiotic with this characteristic. Its antibacterial activity is attributed to its ionophoric action, resulting in the loss of bacterial membrane potential [209]. It was observed that this compound is ineffective against Gram-negative bacteria where the outer membrane appears to block the access of the compound to the cytoplasmic membrane [166].

In the study by Moreira, Aziz and Dick [166] boromycin demonstrated a MIC of 0.08 μM against Mtb H37Rv, evidencing potent antimycobacterial activity. The researchers also evaluated the bactericidal activity, and boromicin demonstrated identical MIC_90_, MBC_99_ and WCC_99_ values of 0.2 μM, both for replicating and non-replicating Mtb bacilli. This implies that boromicin is capable of eradicating not only aerobic growing bacilli cultures but also non-proliferating hypoxic cultures, highlighting its broad action.

To evaluate the selectivity index of boromicin, the researchers performed cytotoxicity analysis against HepG2 and Vero cells. The IC_50_ values obtained were 35.25, and 40 μM, respectively, indicating that boromicin has high selectivity for mycobacterial membranes, with a promising selectivity index, emphasizing its ability to preferentially affect mycobacterial cells compared to host cells. Moreover, to determine the frequency of spontaneous mutations conferring resistance to boromycin, cultures of BCG were exposed to prolonged incubation time with the metabolite. No resistant colonies were observed, demonstrating that the frequency of spontaneous mutations of BCG bacilli resistant to boromicin is very low. In contrast, selection with isoniazid produced resistant strains with a frequency ten times superior, highlighting the effectiveness of boromicin in preventing the development of spontaneous resistance. Thus, boromicin appears to be a highly effective antibiotic, exhibiting a high selectivity index and an extremely low risk of inducing bacterial resistance. These attributes suggest its great potential as an antimicrobial agent in the treatment of TB.

*Lobophorins*. Lobophorins belong to class II spirotetronates, a group of polyketides with complex chemical structures, which comprise more than 70 compounds. These structures have stood out for their expressive biological potential [210, 211].

In the study by Lin et al. [167], four lobophorins (I, F, B, C) (Fig. 7) were tested against Mtb H37Ra, exhibiting moderate MIC values of 2.6 μM, 7.8 μM, 1.3 μM, and 1.4 μM, respectively. In order to evaluate the potential of lobophorins for TB treatment, the authors also assessed the cytotoxic potential of these compounds against the CEM-TART lymphoblastoid cell line. Infortunately, all antimycobacterial active compounds also exhibited strong cytotoxicity.

The authors observed several relevant relationships between chemical structure and biological activity, providing valuable insights for the development of antimycobacterial agents with improved selectivity. The removal of one digitoxose unit, as seen in lobophorin F, resulted in a cytotoxic selectivity greater than 25-fold for human cells compared to Mtb. In contrast, lobophorins B and C demonstrated similar potency toward both cell types, indicating low selectivity. Lobophorin I, however, which contains an α,β-unsaturated aldehyde group, exhibited a more favorable selectivity profile, showing over three times greater activity against Mtb than against human cells. This finding underscores the potential of simple structural modifications in the lobophorin scaffold to generate derivatives with more selective antimicrobial properties.

In the study by Chen et al. [168] three loboforins G, A and B were tested against *M. bovis* BCG and Mtb H37Rv. Against *M. bovis* BCG, the three compounds showed MIC of 1.3 μM, 1.35 μM, and 0.65 μM, respectively. When tested against Mtb H37Rv, the MIC were 26.7 μM, 27.7 μM, and 13.5 μM, respectively. Based on the results obtained, the authors observed that the nitro-sugar moiety plays a fundamental role in enhancing the antimycobacterial activity of lobophorin B, while the modification by acetylation of the hydroxyl group at C-32 in lobophorin G did not result in a significant improvement in activity when compared to lobophorin A. Therefore, these data, together with the biosynthesis studies of lobophorins, indicate the potential for the targeted development of new analogues with optimized bioactivities.

*Steffimycins*. Steffimycin is classified as an atypical anthracycline. This compound exhibits unique structural features that distinguish it from other members of this class, including the presence of a ketone group at C-10, two methoxy groups at C-2 and C-8, absent in compounds such as nogalamycin, aclacinomycin, and daunorubicin, and a neutral deoxy sugar at C-7, replacing the amino sugar commonly found in other anthracyclines [212–214] (Fig. 7).

In the study by Trenado-Uribe et al. [169], steffimycin B was tested against Mtb H37Rv, H37Ra, and RR Mtb, showing MIC values of 13.2 μM, 0.01 μM, and 6.6 μM, respectively. In the study by Koyama et al. [170], steffimycin, 10-dihydrosteffimycin, and 8-demethoxysteffimycin were evaluated against *M. bovis* BCG, *M. avium* complex, *M. intracellulare*, and *M. smegmatis*. Steffimycin showed MIC values of 1.4 μM, 2.7 μM, 0.7 μM, and 2.7 μM, respectively; 10-dihydrosteffimycin exhibited MIC of 21.7 μM, 5.4 μM, 1.4 μM, and 5.4 μM; and 8-demethoxysteffimycin demonstrated MIC of 5.8 μM, 11.5 μM, 0.7 μM, and 5.8 μM. These results indicate moderate antimicrobial activity, with variations suggesting that modifications at C-8 and C-10 significantly influence antimicrobial potency. The compounds also demonstrated moderate cytotoxicity in HeLa cells, with IC₅₀ values of 47.2, 68.4, and 57.0 µg/mL, respectively. Analysis of selectivity based on cytotoxicity and antimycobacterial activity against *M. intracellulare* revealed favorable selectivity ranging from 87.7 to 146.2-fold, surpassing the structurally related daunorubicin, which exhibited higher cytotoxicity and lower selectivity.

In the study by Itaraudom et al. [171], steffimycins C and B were tested against Mtb H37Ra, with MIC values of 0.32 μM and 0.0052 μM, respectively, demonstrating high potency against this strain. The authors also evaluated the cytotoxicity of the compounds and observed relatively low toxicity toward both cancerous and non-cancerous mammalian cells, indicating a potentially favorable safety profile.

*Treponemycin*. Treponemycin is an 18-membered polyketide macrolide antibiotic produced by *Streptomyces* species, exhibiting potent activity against a wide range of bacterial pathogens (Fig. 7). Treponemycin shows antibacterial activity by acting as an inhibitor of threonyl-tRNA synthetase and features a nitrile moiety, a unique functionality in natural products [215–217]. Moreover, the antibiotic demonstrated inhibitory activity against several species of pathogenic bacteria, especially *Treponema hyodysenteriae*, the causative agent of swine dysentery [218].

Treponemycin exhibited moderate activity against Mtb, with a MIC of 8.52 μM [172]. This activity likely occurs because treponemycin interferes with the enzyme threonyl-tRNA synthetase, which is essential for protein synthesis in cells.

*Chrysomycin*. Chrysomycin A is an antibiotic with antitumor properties recovered together with chrysomycin B, as part of a bioactive mixture [219]. Only in 1980 the molecules were separated and their chemical structures properly elucidated [220], and chrysomycin A was later associated with various biological activities [221] (Fig. 7).

Muralikrishnan et al. [173] evaluated the effect of chrysomycin A against Mtb H37Rv. The compound exhibited a MIC of 6.15 μM. However, its biosynthesis via *Streptomyces* fermentation results in low yields, and its mechanism of action is still not fully understood [222, 223].

*Urdamycins.* Belonging to the class of angucycline antibiotics, this category represents a growing group of secondary metabolites derived from microorganisms, particularly from the genus *Streptomyces* [224, 225]. These substances have a characteristic structure composed of a tetracyclic benzoanthracene core, which distinguishes them from anthracyclines. They exhibit a broad spectrum of biological activities and standing out especially for their antitumor potential, in addition to possessing antibacterial, antiviral properties, and enzyme inhibition activities [226] (Fig. 7).

In the study by Supong et al. [174], the compounds urdamycinone E, urdamycinone G, dehydroxyaquayamycin, and urdamycin E showed antimicrobial activity against Mtb with MIC values of 5.88 μM, 24.31 μM, 14.39 μM, and 14.03 μM, respectively. The structural differences influencing the MIC values are mainly related to the presence and position of hydroxyl groups on the anthraquinone ring, the pattern of C-glycosidic bonding, and specific substitutions on the aromatic rings, which alter the compounds interactions with biological targets, affecting the antitubercular activity. The authors also observed that these four substances exhibited cytotoxicity against cancerous cells and non-cancerous cells.

*Kimidinomycin.* Kimidinomycin is a 38-membered macrolide featuring an N-methylguanidyl group at the terminus of the side chain [175] (Fig. 7). Antimycobacterial activity evaluation demonstrated that the compound exhibited MIC values of 23.66 μM against *M. bovis* BCG, 11.83 μM against *M. avium*, 0.74 μM against *M. intracellulare*, and 11.83 μM against *M. smegmatis*. Macrolides are among the oldest and clinically effective antibiotics, targeting the bacterial ribosome by partially blocking the nascent peptide exit tunnel, thereby inhibiting protein synthesis [227].

*Diazaquinomycins.* Diazaquinomycins (DAQs) are secondary metabolites characterized by a 1,8-diazaanthraquinone core, which incorporates two nitrogen atoms into the traditional anthraquinone scaffold, imparting distinct redox properties and electronic characteristics [228]. Variants such as diazaquinomycins H and J are notable for their long lipophilic side chains terminating in an isopropyl group, a structural feature correlated with enhanced antimicrobial potency [228] (Fig. 7).

In the study conducted by Mullowney et al. [176], diazaquinomycins A, J, and H were evaluated against Mtb H37Rv. These compounds exhibited MIC of 0.28 μM (DAQ-A), 0.18 μM (DAQ-J), and 0.10 μM (DAQ-H). Remarkably, they also showed efficacy against MDR strains, while displaying minimal activity against other Gram-positive and Gram-negative bacteria, indicating a selective antimicrobial spectrum [176].

Although DAQ exhibit excellent in vitro activity against Mtb, their clinical application remains limited, primarily due to poor aqueous solubility, which hinders formulation and evaluation in animal models [229]. Nevertheless, recent advances in the heterologous expression of DAQ biosynthetic gene clusters have enabled the production of structural analogues with potentially improved pharmacokinetic profiles [229].

**Fig. 8:**
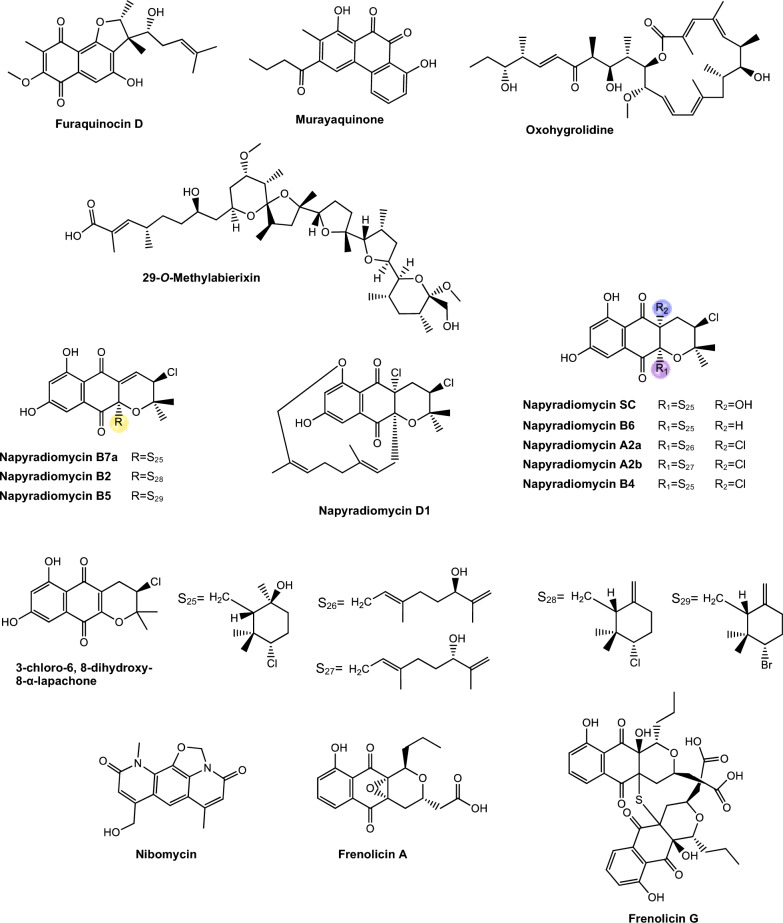
Chemical structures of PKS-derived antimycobacterial metabolites

*Murayaquinone and other compounds*. Murayaquinone is a derivative of naphthoquinone, a class of aromatic compounds characterized by a naphthalene ring bearing two ketone groups at quinonoid positions (Fig. 8). This quinone core imparts redox properties that enable the generation of ROS, which are often associated with antimicrobial and cytotoxic effects [230].

In the study conducted by Bunbamrung et al. [177], murayaquinone was isolated along with four other secondary metabolites: methyl aeruginoate, (R)-desferri-ferrithiocin methyl ester, desferri-ferrithiocin-4-hydroxyphenethyl ester, and furaquinocin D. Among the compounds tested, murayaquinone demonstrated the highest potency, with an MIC below 10 μM. However, it also exhibited significant cytotoxicity against both cancerous and non-cancerous human cell lines, indicating low selectivity for Mtb cells [177]. These findings suggest that murayaquinone holds therapeutic potential as a structural prototype for the development of new antitubercular agents [147, 231].

**Fig. 9:**
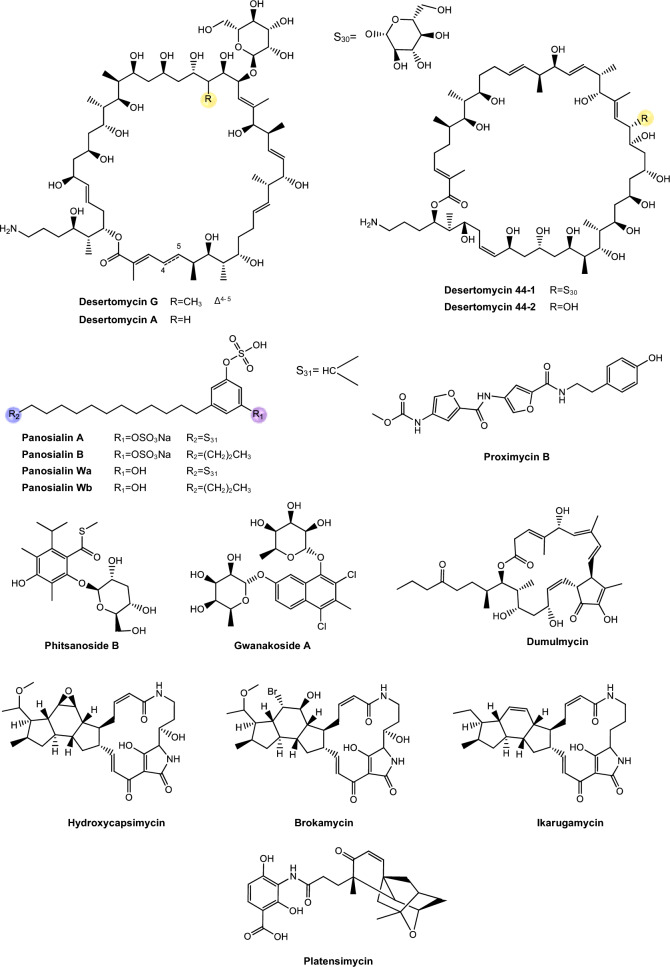
Chemical structures of PKS-derived antimycobacterial metabolites

*Other PKS-derived metabolites*. Several compounds identified in this study showed antimicrobial activity against mycobacteria with MIC values ​​greater than 10 µM, and were considered active, although with limited potency. These includes lincolnenins [162], methyl aerugionate and furaquinocin D [177], oxohygrolidine and methylabierixin [178], napyradiomycins [179], nibomycin [180], frenolicins [181], desertomycins [182, 183], panosialins [184], gwanacoside [185], phitsanoside [186], proximycin [187], dumulmycin [188], hydroxycapsimycin, brokamycin, igakuramycin and platensimycin [189, 190] (Figs. 8, 9). Despite the low activity observed in vitro, these compounds represent valuable PKS-derived scaffolds. This pathway confers a modular and highly diversifiable chemical architecture, which makes these molecules particularly attractive as starting points for rational structural optimization strategies. Through modern medicinal chemistry, it is possible to modify specific functional groups, such as side chains, aromatic systems, and polar regions, aiming to improve pharmacodynamic and pharmacokinetic properties. These natural molecules hold great potential to be optimized into efficient prototypes against TB, contributing to the development of new therapeutic agents in the face of antimicrobial resistance pandemics [232–234].

#### Nucleotide-, aminoglycoside-derived and other antimycobacterial metabolites

Structurally modified nucleosides produced by species of the genus *Streptomyces* exhibit broad applicability in both agricultural and pharmaceutical fields. They function as bioactive molecules whose efficacy and structural diversity stem from their nature as low molecular weight metabolites, which are generated through highly specialized biosynthetic pathways regulated by sophisticated enzymatic systems [235]. As an example, we can mention the puromycin, that causes premature chain termination during translation taking place in the ribosome [236].

On the other hand, aminoglycosides are a class of antibiotics characterized by amino sugar-modified structures, consisting of two or more amino sugars connected by glycosidic linkages to a central hexose ring [237]. Aminoglycosides are primarily obtained through fermentation of *Streptomyces* and *Micromonospora* species [238]. This antibiotic class inhibit protein synthesis by binding to the 30S ribosomal subunit, causing misreading of mRNA and protein misfolding [237]. Aminoglycosides have a gigantic importance in the history of TB treatment, since streptomycin was the first effective antibiotic. In addition, *Streptomyces kanamyceticus* yields kanamycin, which is used for the semi-synthesis of amikacin, which is still used for the treatment of resistant forms of TB.

Table 3 presents nucleotide, aminoglycoside and miscellaneous metabolites isolated from different Actinomycetota species, along with their respective MIC values against mycobacterial strains.

**Fig. 10:**
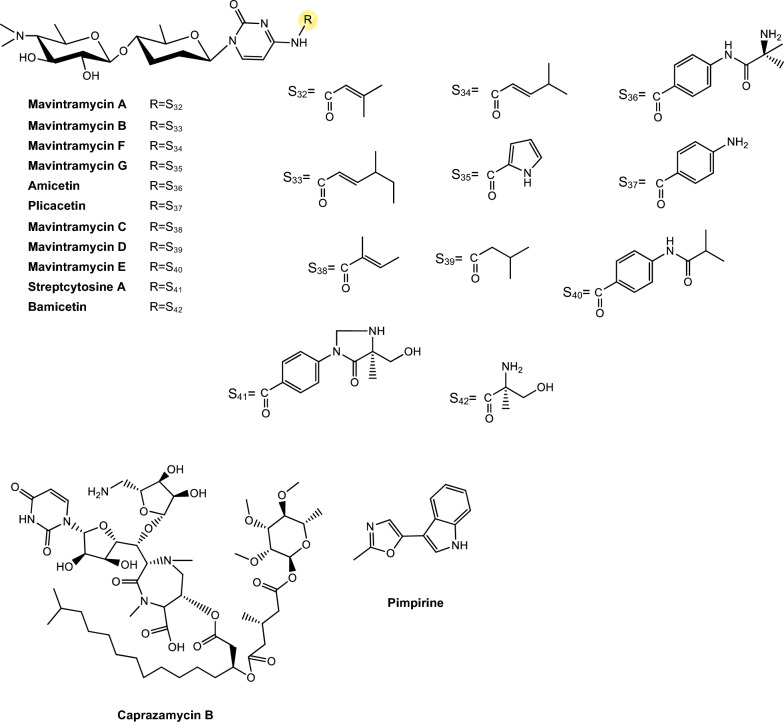
Chemical structures nucleotide-derived antimycobacterial metabolites

*Mavintramycins*. Mavitramycins These consisted of common cytosine, amosamine, and amicetose moieties and a diverse R moiety [239] (Fig. 10). In the study conducted by Hosoda et al. [239] mavintramycins A-G were evaluated for their antimycobacterial activity against strains of the *M. avium* complex, *M. smegmatis*, and *M. bovis*, demonstrating MIC ranging from 0.79 to 104.11 μM. Among them, mavintramycin A displayed the highest potency, with MIC of 0.81 μM and 1.62 μM against *M. avium* and *M. intracellulare*, respectively.

In addition to mavintramycins, the authors isolated amicetin (Fig. 10), a nucleoside antibiotic of the aminohexopyranose class. This compound is part of a broader family that includes bamicetin, oxamicetin, SF2457, and cytimidine, all sharing structural motifs involving a modified hexose linked to a nitrogenous base [246]. Amicetin exhibited moderate to high antimycobacterial activity, with MIC values ranging from 0.63 to 5.04 μM against the tested strains.

Another compound identified was plicacetin (Fig. 10), a nucleoside antibiotic with previously documented activity against Mtb [247]. When tested under the same conditions, plicacetin showed consistent MIC of 6.03 μM, except for *M. bovis*, for which the MIC was 1.51 μM. When comparing the compounds, the authors observed that mavintramycin A was more potent than amicetin against *M. avium* and *M. intracellulare*. This suggests that the presence of a ρ-aminobenzoic acid group at the R position is not essential for biological activity, and that structural variations at this site strongly influence antimicrobial potential [239].

Mechanistic investigations revealed that mavintramycin A exerts its action by binding to the 23S ribosomal RNA, thereby inhibiting protein synthesis and secondarily impairing DNA replication. Based on these promising results, the authors advanced to in vivo infection models, demonstrating that the compound was effective both in *M. smegmatis*-infected silkworms and in a murine model of *M. avium* infection, significantly reducing bacterial loads in host tissues [239].

Taken together, the findings suggest that mavintramycin A possesses relevant therapeutic potential, especially for the treatment of TB and infections caused by *M. avium* complex. Its use in combination with conventional drugs could enhance antimicrobial efficacy and help combat drug-resistant strains.

*Caprazamycins*. Caprazamycins belong to the group of liponucleoside antibiotics [248]. Their molecular architecture is centered around the core of the compound ( +)-caprazol, composed of 5'-glycyluridine, 5-amino-D-ribose, and a methylated diazepanone ring (Fig. 10). It inhibits the enzyme phospho-MurNAc-pentapeptide transferase, responsible for catalyzing the transfer of the phospho-MurNAc-pentapeptide from UDP-MurNAc-pentapeptide to undecaprenyl phosphate (C55-P), a crucial step in bacterial cell wall biosynthesis [249].

Igarashi et al. [240] evaluated caprazamycin B against Mtb H37Rv, *M. bovis*, drug-susceptible Mtb and MDR Mtb, obtaining MIC values of 2.73 μM, 2.73 μM, 5.45 μM, and 5.45 μM, respectively. Moreover, the researchers reported that this compound did not exhibit significant toxic effects in mice or in genotoxicity and cytotoxicity assays, thus suggesting it as a promising candidate for the development of new anti-TB drugs.

**Fig. 11:**
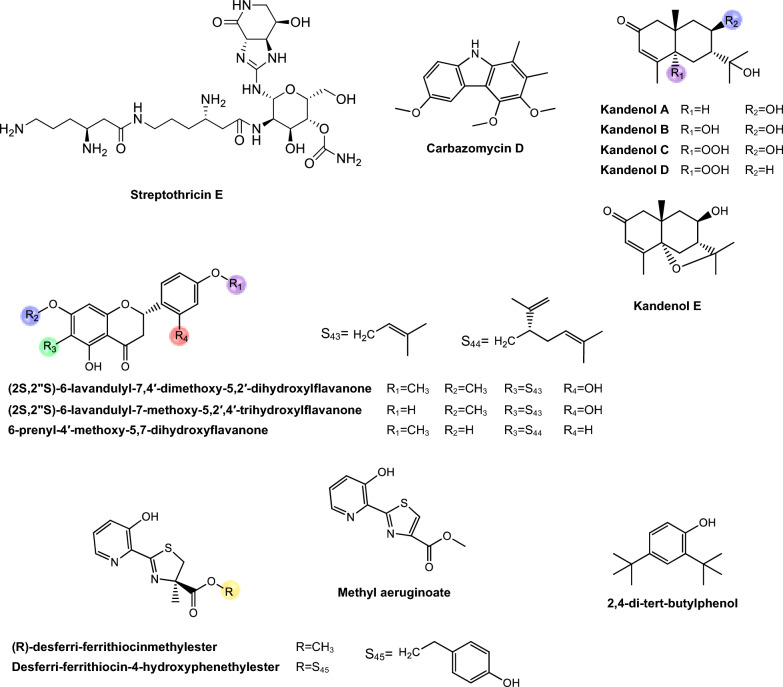
Chemical structures of antimycobacterial metabolites

Other metabolites. Streptothricin is one of the earliest antibiotics isolated from environmental bacteria, effective against both Gram-positive and Gram-negative species. Structurally, it comprises a streptolidine lactam ring, the sugar gulosamine, and a β-lysine polymer varying from one to seven units [250] (Fig. 11). Its mechanism involves binding to the 30S ribosomal subunit, inhibiting protein synthesis. Initially promising due to its broad-spectrum activity, streptothricin’s clinical use was limited by reversible nephrotoxicity observed in early trials [251]. Currently, research focuses on developing derivatives with improved safety profiles to address antibiotic resistance challenges. Thus, streptothricin remains an important scaffold for antibiotic development due to its unique mechanism and structural diversity [241, 250, 251]. Gan et al. [241] tested streptothricin E (Fig. 11) against Mtb H37Rv. This compound exhibited a MIC of 1.59 μM. However, in a preliminary clinical study, the use of this metabolite led to reversible renal toxicity, which halted its progress as a potential therapeutic agent [251].

Streptcytosine A, plicacetin, bamicetin, carbazamycin D, and pimprinine (Fig. 11) were evaluated against *M. smegmatis*. The compounds exhibited MIC of 50.74 μM, 61.82 μM, 26.46 μM, 87.61 μM, and 126.12 μM, respectively, with bamicetin standing out as approximately twice as active as the other tested compounds.

Kandenols are eudesmane-type sesquiterpenes (Fig. 11) that are common in plants and are involved in ecological defense. However, their occurrence in bacteria is considered extremely rare [243]. Ding et al. [243] isolated five new kandelons (A-E), representing the first known examples of eudesmane-type sesquiterpenes derived from a bacterial source. When tested against *M. vaccae*, the kandenols showed MIC of 43.96 μM-93.16 μM. Additionally, the compounds were evaluated for cytotoxicity against twelve human cancer cell lines and did not exhibit significant cytotoxic activity.

(2S,2″S)-6-lavandulyl-7,4′-dimethoxy-5,2′-dihydroxyflavanones, (2S,2″S)-6-lavandulyl-7-methoxy-5,2′,4′-trihydroxyflavanones, and 6-prenyl-4′-methoxy-5,7-dihydroxyflavanones (Fig. 11), three lavandulylated flavonoids were evaluated for against Mtb H37Rv, exhibiting MIC of 106.15 μM, 13.69 μM, and 31.32 μM, respectively [244]. Additionally, cytotoxicity assays performed on human cells showed no significant toxic effects. 2,4-Di-tert-butylphenol (Fig. 11) was identified by Kaari et al. [245] and tested against an MDR Mtb, exhibiting a MIC of 242.37 μM, as well as the standard Mtb H37Rv strain, with a MIC of 48.47 μM. The results underscore the potential of these compounds as a promisings candidates for the development of new drugs for future therapeutic applications against TB.

## Conclusion remarks

Compared to other bacterial infectious diseases, treatment of TB represents a significant challenge due to the slow growth of Mtb and its ability to evade the host immune system, which prolongs therapy and hinders complete eradication of the pathogen. This scenario becomes even more complex with the emergence of MDR and XDR strains, which compromise the efficacy of current treatment regimens [5]. Beyond the direct impact on public health, antimicrobial resistance causes substantial economic losses, with projections indicating that, by 2050, drug-resistant TB could result in global losses of approximately 16.7 trillion dollars [62, 252].

Given this problem, there is an urgent need to develop new antitubercular drugs that act through mechanisms distinct from those currently available, with lower toxicity, higher selectivity, shorter, and safer therapeutic regimens. In this context, natural products emerge as a promising source of compounds with therapeutic potential due to their structural diversity and recognized biological activity [1, 253, 254].

In this review, 171 secondary metabolites produced by Actinomycetota species with antimycobacterial activity were analyzed, particularly against Mtb strains. Among the most potent compounds, with MIC in the submicromolar range, are the compounds steffimycin B (0.0052 μM), ilamycin J (0.0096 μM), nosiheptide (0.01 (0.03 μM)) actinomycins C2 and C3 (0.03 μM), rufomycin NBZ8 (0.030 μM), lassomycin (0.07 μM) and boromycin (0.08 μM). These compounds represent highly promising candidates for the development of new antitubercular drugs. Additionally, some of these substances also demonstrated activity against resistant Mtb strains, which is particularly relevant given the difficulty of treating MDR and XDR strains.

It is worth noting that natural metabolites derived from microorganisms play a crucial role in the discovery of new drug candidates, especially for diseases that still have large therapeutic gaps, such as TB. Recent studies have shown that biosynthetic gene clusters present in Actinobacteria encode a wide chemical diversity, and modern approaches such as genome mining, genomic mapping, and chemical dereplication have accelerated the discovery of new bioactive molecules, including those from underexplored niches such as marine environments and symbiotic associations [18, 19, 255].

Therefore, the data gathered in this review reinforce that Actinobacteria constitute a promising and still underexploited source of antimycobacterial compounds with clinical potential. Advances in understanding their mechanisms of action, combined with structural optimization and in-depth in vivo evaluations, may significantly contribute to the incorporation of new molecules into the therapeutic arsenal against TB, especially against resistant forms.

## Data Availability

Not applicable.
